# Structuring Variability in Human Gait Datasets: A Covariate-Centered Taxonomy and Systematic Review of Image- and Depth-Based Collections

**DOI:** 10.3390/jimaging12070334

**Published:** 2026-07-22

**Authors:** João Ferreira Nunes, Pedro Miguel Moreira, João Manuel R. S. Tavares

**Affiliations:** 1 ADiT-Lab, Instituto Politécnico de Viana do Castelo, 4900-347 Viana do Castelo, Portugal; pmoreira@estg.ipvc.pt; 2 Instituto de Ciência e Inovação em Engenharia Mecânica e Engenharia Industrial, Departamento de Engenharia Mecânica, Faculdade de Engenharia, Universidade do Porto, 4200-465 Porto, Portugal; tavares@fe.up.pt

**Keywords:** gait analysis, dataset review, RGB, RGB-D, depth, taxonomy, covariates, benchmarking, computer vision

## Abstract

Human gait datasets play a central role in the development and evaluation of computer vision models. However, the current dataset landscape remains highly heterogeneous, with inconsistent reporting of acquisition conditions, user variability, and sensing configurations, which limits reproducibility and hinders principled cross-dataset comparability. In this work, we propose a covariate-centered, modality-agnostic taxonomy for gait datasets, explicitly structuring variability across scene-level, user-level, and sensor-level factors. The proposed framework enables consistent characterization of datasets through a standardized set of covariates (A–R), bridging differences across application domains and sensing modalities. Following a systematic review protocol aligned with PRISMA 2020, we analyze 47 publicly available image- and depth-based human gait datasets spanning healthcare, biometric, and attribute-recognition application domains. Using the proposed taxonomy, we derive a quantitative analysis of covariate coverage, revealing systematic biases in current dataset design.

## 1. Introduction

Gait analysis is the scientific study of human walking, with applications ranging from clinical assessment and rehabilitation monitoring to person recognition at a distance. In the domain of computer vision, the analysis of human gait is predominantly supported by image-based and depth-based sensing methodologies. These include RGB video, depth and RGB-D data, infrared imagery, silhouettes, pose/keypoints, skeleton sequences, and human parsing representations. Recent survey literature attests to the broad range of applications and the rapid methodological progress enabled by deep learning, covering gait recognition pipelines, body representations, temporal modeling strategies, neural architectures, and evaluation protocols across benchmark datasets [1,2,3,4]. Vision-sensor reviews in human gait analysis have also highlighted the increasing adoption of non-invasive depth cameras and learning-based approaches for extracting clinically relevant gait information [5,6].

Despite this progress, empirical performance and generalization remain predominantly influenced by the structure of the dataset. Variability is introduced at multiple stages of the observation pipeline, including environmental and scene-level conditions, such as viewpoint configuration, illumination, background complexity, and scene geometry; user-level factors, such as gait condition, walking speed, clothing, carried objects, footwear, demographic attributes, and clinical status; and sensor-level factors, such as acquisition modality, frame rate, spatial resolution, camera configuration, calibration, and synchronization. These sources of variability, commonly referred to as covariates, have a substantial influence on the formation of silhouettes, the stability of pose estimation, the consistency of temporal modeling, and the comparability of experimental results across datasets.

A recurring theme in the literature is that covariates can significantly degrade recognition performance and compromise cross-dataset comparability. This motivates the need for covariate-aware evaluation and careful interpretation of protocol labels such as “cross-view”, “cross-clothes”, “cross-speed”, or “in-the-wild” [7,8,9,10]. In the context of deep learning, this issue is especially relevant because models may achieve high performance under controlled acquisition conditions while failing to generalize to unseen viewpoints, appearance changes, occlusions, walking conditions, or sensor configurations. Consequently, dataset variability directly affects benchmark design, model evaluation, domain transfer, and the interpretation of robustness claims.

Previous surveys and taxonomies have made important contributions to the organization of gait-analysis research. Some works classify gait recognition methods according to body representation, temporal representation, feature representation, and neural architecture; others focus on deep learning strategies, intra-class covariates, benchmark protocols, person re-identification, or vision-based sensor systems. In these works, datasets are commonly discussed as experimental resources, benchmark sources, or application-specific collections. However, the structure of the datasets themselves, namely how they encode, document, omit, and combine sources of variability, is rarely treated as the primary object of analysis. This distinction is important because dataset-level variability directly conditions protocol design, cross-dataset comparability, and the interpretation of robustness claims. The present work therefore differs from previous surveys by shifting the focus from algorithms, tasks, or sensing technologies to the systematic characterization of dataset variability through a covariate-centered taxonomy. Table 1 summarizes this positioning by comparing the proposed taxonomy with representative surveys and classification frameworks in gait analysis.

As shown in Table 1, previous works provide valuable method-oriented, task-oriented, covariate-handling-oriented, or modality/sensor-oriented perspectives. In contrast, the present article focuses on dataset structure itself by examining how publicly available gait datasets encode, document, omit, and combine sources of variability. This article addresses this gap by proposing a covariate-centered and modality-agnostic framework for the structured characterization of publicly available image- and depth-based human gait datasets. The proposed taxonomy treats dataset variability as the primary object of analysis and organizes covariates into scene-level, user-level, and sensor-level dimensions. This structure enables datasets from different application domains and sensing modalities to be described, compared, and interpreted using a common analytical framework. To the best of our knowledge, this is one of the first systematic attempts to characterize publicly available image- and depth-based gait datasets through a unified taxonomy centered on covariate coverage, documentation quality, and benchmark utility.

The review process was guided by PRISMA 2020 (Supplementary Materials), the *Preferred Reporting Items for Systematic reviews and Meta-Analyses* [11], a reporting guideline intended to improve the transparency, completeness, and reproducibility of systematic reviews. The reviewed datasets span the domains of healthcare, biometric, and attribute-recognition, allowing the proposed taxonomy to be used not only as a descriptive classification tool but also as a basis for identifying dataset-design biases, documentation gaps, and limitations affecting model robustness and reproducible evaluation.

### Contributions

The main contributions of this work are fourfold:

- We propose a covariate-centered and modality-agnostic taxonomy for publicly available image- and depth-based gait datasets, organizing dataset variability into scene-level, user-level, and sensor-level dimensions.
- We conduct a PRISMA-guided systematic review of 47 publicly available gait datasets across healthcare-oriented, biometric-oriented, and attribute-recognition domains, documenting the dataset selection process, eligibility criteria, and extracted metadata to support reproducibility.
- We instantiate the proposed taxonomy across the reviewed datasets, providing a structured comparative analysis of dataset scale, acquisition settings, sensing modalities, sequence characteristics, annotation richness, covariate coverage, and documentation quality.
- We synthesize the main limitations of the current dataset landscape and show how the proposed taxonomy can support dataset selection, covariate-aware benchmark design, model evaluation, and future dataset reporting practices.

## 2. Review Methodology

**Figure 1:**
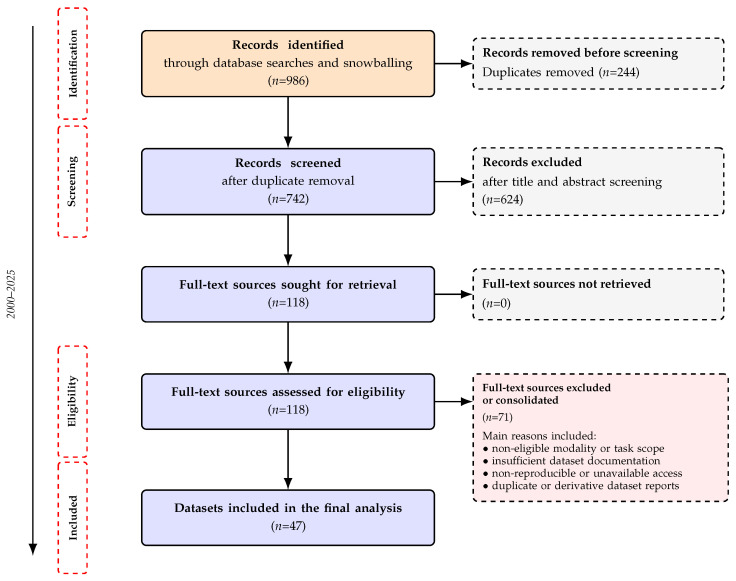
Adapted PRISMA 2020-style flow diagram for the dataset selection process.

This study adopts a structured and reproducible review protocol aligned with PRISMA 2020, the *Preferred Reporting Items for Systematic reviews and Meta-Analyses* framework [11], and adapted to dataset-oriented research in computer vision. The review protocol was registered in the Open Science Framework (OSF) under DOI (https://doi.org/10.17605/OSF.IO/MSNZY) [12]. The protocol documents the research questions, eligibility logic, and planned dataset-oriented synthesis, supporting transparency in dataset identification, screening, eligibility assessment, inclusion, and taxonomy-based coding while accounting for the heterogeneity of gait data acquisition and reporting practices. The overall selection workflow is summarized in Figure 1.

Unlike conventional systematic reviews in which the final unit of analysis is typically a publication, the final unit of analysis in this review is the dataset. Therefore, when multiple records described the same dataset, the most complete dataset paper, official dataset page, repository, or documentation source was used as the primary source for data extraction, while complementary records were consulted when they provided additional acquisition, annotation, access, or protocol details.

### 2.1. Research Questions

The review was guided by a set of research questions documented in the OSF registration. These questions were formulated to delimit the scope of the review, structure the dataset selection process, and align the subsequent analysis with the proposed covariate-centered taxonomy:

- **RQ1.** Which publicly accessible image- and depth-based datasets have been made available for human gait analysis, gait recognition, healthcare-oriented gait assessment, and gait-related attribute recognition?
- **RQ2.** What scene-level, user-level, and sensor-level sources of variability are explicitly represented or reported in these datasets?
- **RQ3.** How are gait datasets distributed across application domains, namely healthcare-oriented, biometric-oriented, and attribute-recognition contexts?
- **RQ4.** Which covariates are most frequently represented, under-represented, or insufficiently documented across the included datasets?
- **RQ5.** How can a covariate-centered taxonomy support a more reproducible and comparable characterization of heterogeneous human gait datasets?

RQ1 guided the identification and inclusion of publicly accessible datasets. RQ2 and RQ4 guided the extraction, coding, and synthesis of covariate information. RQ3 supported the organization of datasets by application domain. RQ5 guided the interpretation of the practical role of the taxonomy in dataset comparison, benchmark design, and reproducible model evaluation.

### 2.2. Search Strategy

A comprehensive search was conducted to identify publicly accessible image- and depth-based human gait datasets reported in the literature. The search covered major scientific indexing platforms, including IEEE Xplore, Scopus, and Web of Science. Google Scholar was used as a complementary source rather than as a primary indexing database, mainly to identify dataset webpages, repositories, technical reports, and records not consistently indexed in bibliographic databases. The publication window was restricted to 2000–2025, corresponding to the main period of development of vision-based gait analysis datasets. This interval also captures the transition from early video- and silhouette-based gait datasets to more recent collections involving depth sensors, RGB-D acquisition, large-scale unconstrained video, and richer annotation protocols. The search strategy was therefore designed to balance bibliographic coverage with practical dataset traceability, ensuring that both peer-reviewed publications and associated access points were considered.

Search queries were designed to capture both dataset-centric and application-oriented terminology. The general Boolean structure was

(“gait dataset” OR “gait database” OR “human gait recognition dataset” OR “gait analysis dataset” OR “walking dataset”) AND (RGB OR depth OR RGB-D OR silhouette OR video OR vision OR image)

To improve coverage and reduce the risk of missing datasets due to inconsistent indexing, additional query variants were used with terms related to application domains, sensing modalities, and covariates:

(“gait recognition” OR “gait analysis” OR “human walking”) AND (dataset OR database OR benchmark) AND (view OR clothing OR carrying OR covariate OR depth OR RGB-D OR silhouette)

The same conceptual query structure was adapted to the syntax and search interface of each source. In Google Scholar, searches were used only as a complementary strategy to locate dataset access pages, repositories, and grey literature associated with candidate datasets identified through database searches or snowballing.

The search was complemented by backward and forward snowballing. Backward snowballing involved inspecting the reference lists of recent surveys, benchmark papers, and dataset publications. Forward snowballing involved identifying later works that cited key dataset papers and checking whether they introduced new datasets or provided missing information about existing ones. Official dataset webpages, institutional repositories, GitHub repositories, and dataset hosting platforms were also inspected when they were referenced by the corresponding publications.

### 2.3. Eligibility Criteria

Datasets were selected based on predefined inclusion and exclusion criteria to ensure relevance, accessibility, and compatibility with the proposed covariate-centered taxonomy.

**Inclusion criteria:**

- **Visual modality:** the dataset must include RGB, video, silhouette-based, depth, RGB-D, infrared, skeleton, pose/keypoint, or human-parsing data derived from visual sensing;
- **Task relevance:** the dataset must contain human gait or walking sequences as a primary component;
- **Public availability:** the dataset must be publicly accessible through direct download, institutional repository, project webpage, GitHub repository, or request-based access under standard data-use conditions;
- **Dataset documentation:** sufficient information must be available to extract at least the core dataset descriptors, including acquisition modality, number of subjects or sequences, acquisition setting, and relevant covariates;
- **Human subjects:** the dataset must involve human participants rather than simulated, synthetic, or non-human motion data.

**Exclusion criteria:**

- Datasets based exclusively on non-visual modalities, such as inertial, pressure, force-plate, electromyography, or wearable-sensor data;
- Datasets in which gait or walking is only incidental and not a primary component of the acquisition protocol;
- Datasets with inaccessible, discontinued, private, or non-reproducible access conditions;
- Datasets lacking essential acquisition, modality, or annotation metadata required for taxonomy coding;
- Duplicate records, derivative reports, or subsets that did not introduce additional subjects, modalities, covariates, annotations, or acquisition conditions beyond a previously included dataset;
- Datasets containing only synthetic, simulated, or avatar-based walking data.

Preference was given to marker-free capture setups because the scope of this review is image- and depth-based gait analysis in computer vision. However, datasets including visual data together with other modalities were retained when the visual component was central to the dataset and could be independently characterized.

### 2.4. Screening and Selection Process

The selection procedure followed a multi-stage pipeline aligned with PRISMA principles. Records were first identified through database queries and complementary snowballing. After duplicate removal, titles and abstracts were screened to discard clearly irrelevant records. The remaining records underwent full-text assessment against the eligibility criteria.

At the full-text stage, records were excluded if they did not meet the modality requirements, did not provide reproducible access to the dataset, lacked sufficient documentation for covariate extraction, or corresponded to derivative reports of previously included datasets without additional dataset-level information. When several records referred to the same dataset, they were consolidated into a single dataset entry. In such cases, the official dataset page or the most complete dataset publication was used as the primary source, and additional records were used only to complement missing metadata.

Eligibility decisions were based on triangulation between the dataset paper, official dataset documentation, access page, and, when available, repository metadata. Ambiguous cases were resolved by rechecking the original source, dataset webpage, and associated publications.

In total, 986 records were identified through database searches and snowballing. After duplicate removal, 742 records remained for title and abstract screening. Following this screening stage, 118 full-text articles or dataset reports were assessed for eligibility. After applying the inclusion and exclusion criteria and consolidating multiple records describing the same dataset, 47 datasets were included in the final analysis. Because the unit of analysis was the dataset rather than the individual publication, full-text sources that did not result in a distinct dataset entry were treated as either excluded or consolidated, depending on whether they failed the eligibility criteria or described a dataset already represented by a more complete source. The overall selection process, including duplicate removal, title and abstract screening, full-text eligibility assessment, source consolidation, and final dataset inclusion, is summarized in Figure 1.

### 2.5. Data Extraction and Taxonomy Coding

For each included dataset, information was extracted using a structured coding template designed to support comparison across application domains, sensing modalities, and acquisition protocols. The extracted information included dataset name, year of publication or release, application domain, access mode, number of subjects, number of sequences or samples, acquisition environment, sensor modality, spatial and temporal resolution, camera configuration, walking protocol, view configuration, user-related factors, annotation types, and reported covariates.

Each dataset was then coded according to the proposed taxonomy. The taxonomy groups covariates into three main dimensions: scene-level covariates, user-level covariates, and sensor-level covariates. Scene-level covariates describe the acquisition environment and external observation conditions; user-level covariates describe human-related variability, including gait condition, appearance, carried objects, demographic characteristics, and clinical information; and sensor-level covariates describe acquisition modality, camera setup, synchronization, and signal formation properties.

Covariate values were extracted only when they were explicitly reported in the dataset publication, official documentation, repository, or access page. When a covariate appeared to be present in the acquisition protocol but was not explicitly documented or could not be reliably inferred, it was coded as not reported. This conservative strategy was adopted to avoid overestimating dataset coverage and to distinguish between covariates that are experimentally controlled, covariates that are naturally present but undocumented, and covariates that are explicitly annotated.

### 2.6. Dataset Quality, Access, and Documentation Assessment

In addition to covariate coverage, each dataset was examined in terms of documentation quality and practical reusability. This assessment considered whether the dataset provides stable public access, clear access conditions, licensing or data-use information, sufficient acquisition metadata, annotation descriptions, demographic information, covariate labels, and recommended evaluation protocols.

This quality-oriented assessment was qualitative and descriptive rather than score-based. It was not intended to rank datasets since datasets differ substantially in purpose, scale, application domain, ethical constraints, and intended use. Instead, it was used to identify recurring documentation and reproducibility issues that affect cross-dataset comparison and benchmark design. Particular attention was given to public availability, annotation traceability and reported annotation procedures, balance across identities, clinical groups, attributes, and covariate conditions, demographic balance, licensing clarity, and long-term accessibility.

## 3. Systematic Review by Main Area of Use

To reflect the prevailing evaluation practice and to streamline navigation, the review is organized by *main area of use*. Each dataset is assigned to a single main area of use according to its primary evaluation objective, while acknowledging that some datasets may support secondary tasks. The three areas considered are: (i) healthcare-oriented datasets, where clinical labels, pathology simulation, monitoring, or event annotations are central; (ii) biometric-oriented datasets, designed for identification/verification and surveillance-style evaluation, typically emphasizing subject scale, multi-view geometry, and covariate robustness; and (iii) attribute-recognition datasets, targeting soft-biometric or behavioral attributes with multi-label or attribute-aware annotations. The resulting classification comprises 11 healthcare-oriented datasets, 33 biometric-oriented datasets, and 3 attribute-recognition datasets.

**Figure 2:**
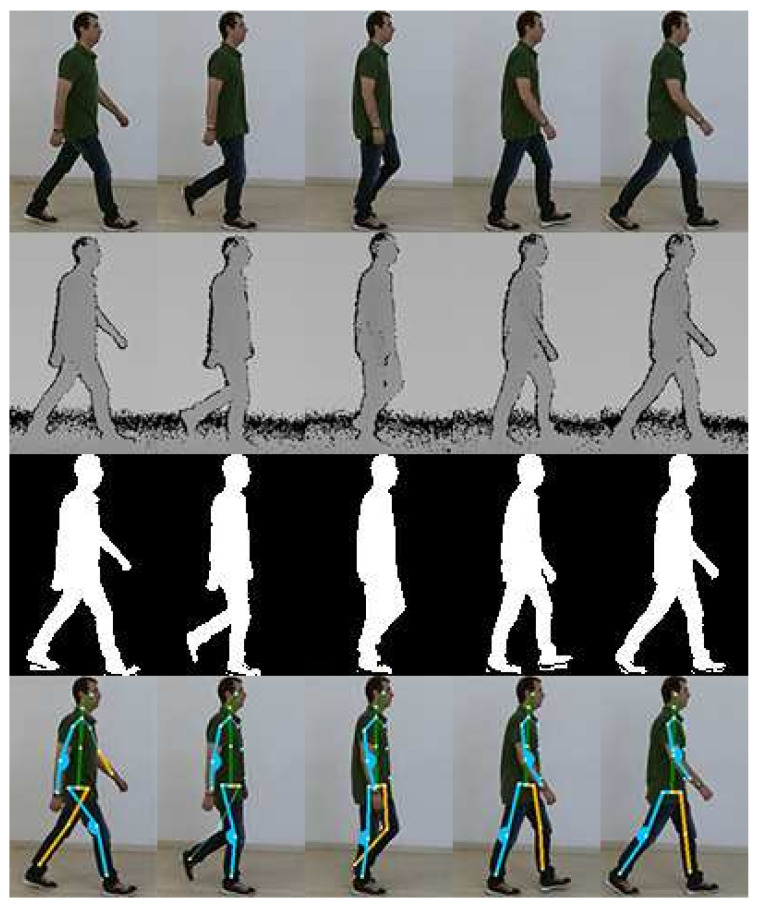
Representative gait data modalities frequently encountered in image- and depth-based datasets, including RGB, depth, silhouette, and pose/skeleton representations. Author-generated illustration based on [13].

Figure 2 illustrates representative data modalities commonly encountered in image- and depth-based gait datasets, including RGB frames, depth, silhouettes, and pose/skeleton representations. The coexistence of heterogeneous modalities motivates the modality-agnostic structural taxonomy introduced in Section 4.

The following subsections discuss datasets within each main area of use, emphasizing chronological evolution in dataset scale, covariate richness, sensing modalities, and annotation depth. Detailed structural characterization using normalized covariate values is provided in the taxonomy-aligned tables in Section 5.

### 3.1. Healthcare-Oriented Datasets

Healthcare-oriented datasets are primarily designed for clinical assessment, rehabilitation monitoring, pathology screening, or the analysis of abnormal locomotion patterns. The dataset scale in this domain ranges from small-scale controlled collections, such as GAIT-IST [14], INIT [15], SPHERE [16], and GAIT-IT [17], to medium- and large-scale resources such as KOA-PD-NM [18], Health&Gait [19], and Scoliosis1K [20].

In terms of sequence characteristics, these datasets are predominantly acquired in indoor and controlled settings, including laboratories, clinical rooms, corridors, treadmill setups, and stair-based environments. Most rely on short, task-specific walking trials, such as straight overground walking, bidirectional walking, treadmill-constrained walking, stair ascent, or assisted pathological walking. Single-session acquisition is common, although some datasets extend the temporal scope through longer collection periods or repeated acquisition campaigns.

The main emphasis in this domain is user-level locomotion variability. Datasets commonly distinguish normal walking from pathological, simulated, asymmetric, prosthetic, or clinically relevant gait patterns, while appearance-related covariates such as clothing, carried objects, and load are usually constrained. Sensor configurations are generally simpler than in biometric-oriented datasets, with single-view RGB, depth/RGB-D, silhouette, and keypoint-based representations being frequent.

### 3.2. Biometric-Oriented Datasets

Biometric-oriented datasets are primarily designed for person identification and verification, typically under controlled, surveillance-inspired, or in-the-wild evaluation scenarios. This domain shows the widest variation in scale, ranging from small controlled datasets such as CASIA-A [21], MoBo [22], GRIDDS [13], and KY4D [23,24], to medium- and large-scale benchmarks such as CASIA-B [25], CASIA-E [26], CCGR [8], and SUSTech1K [27]. Very large-scale resources, including GREW [28], OU-LP-Bag [29], Gait3D-Parsing [30], and the OU-MVLP family [31,32,33,34], reflect the increasing emphasis on large population size and unconstrained or multi-view evaluation.

In terms of sequence characteristics, biometric-oriented datasets range from highly controlled walking trials to complex real-world acquisition. Earlier datasets often rely on straight trajectories, treadmill-constrained walking, or fixed multi-camera setups, whereas more recent collections increasingly include outdoor scenes, multi-session acquisition, unconstrained trajectories, occlusion, or surveillance-style viewpoints. Examples include CASIA-B [25] for controlled cross-view and appearance variation, OU-ISIR treadmill datasets [35,36,37] for speed- and treadmill-based evaluation, and GREW [28] or Gait3D-Parsing [30] for in-the-wild recognition.

The main emphasis in this domain is robustness to identity-preserving variability, including viewpoint, clothing, carrying condition, walking speed, occlusion, and environmental changes. Sensor configurations and data modalities are also heterogeneous, spanning RGB video, silhouettes, gait templates, depth/RGB-D, infrared, keypoints, optical flow, point clouds, parsing, and 3D representations.

### 3.3. Attribute-Recognition Datasets

Attribute-recognition datasets focus on the estimation of soft-biometric, demographic, appearance-related, or behavioral attributes from gait data. This domain is smaller in terms of the number of available datasets but heterogeneous in scale: MA-Gait [38] is a small- to medium-scale multi-view collection, RA-GAR [39] represents a medium-scale outdoor dataset with richer gait-attribute annotations, and OU-LP-Age [40] is a very large-scale dataset primarily oriented toward age-related gait analysis.

In terms of sequence characteristics, these datasets include both controlled indoor protocols and outdoor acquisition. MA-Gait [38] and OU-LP-Age [40] use controlled indoor straight-walking protocols, whereas RA-GAR [39] extends attribute recognition toward outdoor acquisition with natural illumination changes and real-world backgrounds. The main emphasis is user-level semantic annotation, including age, sex, gait attributes, appearance-related descriptors, clothing variation, and carrying-related information.

Sensor configurations include both single-view and multi-view acquisition, with modalities such as GEI, silhouettes, 2D keypoints, and 3D keypoints. Overall, attribute-recognition datasets provide richer semantic annotations than most biometric-oriented collections, but the domain remains comparatively limited in dataset availability, temporal variation, and cross-dataset standardization.

## 4. Covariate Taxonomy Framework

### 4.1. Motivation and Covariate-Centered Design Rationale

As gait datasets have evolved, their documentation has become increasingly heterogeneous. Collections are often characterized according to sensing modality, application domain, or isolated experimental factors such as viewpoint or clothing variation. Although this diversity facilitates targeted experimentation, it also complicates principled cross-dataset comparison. Evaluation protocols labeled as “cross-view” or “cross-clothes” may obscure substantial differences in acquisition geometry, illumination conditions, synchronization strategies, and demographic composition.

A structural taxonomy is therefore required to (i) separate the dominant sources of variability, (ii) formalize the level at which each attribute varies, and (iii) enable consistent mapping between dataset properties and evaluation protocols. Unlike action-centered or modality-centered classifications [41,42], the present proposal adopts a strictly *covariate-centered* and acquisition-aware perspective. Variability is organized according to its origin within the observation pipeline and its impact on the observable representation of gait.

In this work, a *covariate* is defined as any dataset-reported factor that can condition the observable appearance, geometric structure, temporal dynamics, or signal formation of gait, and that may influence dataset comparison, benchmark design, or model evaluation. The role of a factor is task-dependent: the same variable may function as a target label in one setting, as descriptive metadata in another, or as a covariate when it is used to stratify evaluation, define protocol variation, or assess robustness. For example, age may be the prediction target in age-estimation datasets, demographic metadata in biometric datasets, or a stratification covariate in cross-age evaluation. Similarly, clinical status may be a target label in healthcare-oriented datasets but a covariate when assessing robustness across populations. Covariates may therefore be actively varied by design, naturally present in the dataset population, or held constant as controlled acquisition parameters; in all cases, their status should be explicitly documented to support reproducibility and fair evaluation.

In order to preserve analytical clarity while maintaining conceptual economy, the covariates are organized into three analytically distinct, origin-based coding dimensions: scene-level, user-level, and sensor-level. These correspond to the dominant sources of variability in vision-, depth-, and multimodal gait datasets: the physical environment, the participant, and the acquisition hardware.

### 4.2. Formal Definition

Let the complete covariate space be defined as

$$S=S_{\mathrm{scene}}\cup S_{\mathrm{user}}\cup S_{\mathrm{sensor}},$$

where each subset corresponds to a distinct stage of the acquisition and observation process. The subsets are defined as mutually exclusive:

$$S_{\mathrm{scene}}\cap S_{\mathrm{user}}=⌀,\;S_{\mathrm{scene}}\cap S_{\mathrm{sensor}}=⌀,\;S_{\mathrm{user}}\cap S_{\mathrm{sensor}}=⌀.$$

The mutual exclusivity of the three subsets should be understood as a coding principle rather than as a claim that covariates have independent effects on the observed gait signal. Each attribute is assigned to the domain corresponding to its primary source of variability: environmental and contextual conditions are encoded as scene-level covariates, participant-related factors are encoded as user-level covariates, and acquisition or signal-formation properties are encoded as sensor-level covariates. Effects may propagate across domains; however, the taxonomy encodes the origin of the variability rather than all subsequent consequences.

For each dataset (*D*), the taxonomy defines a structured covariate profile:

$$\phi \left(D\right)=v_{1},v_{2},\ldots ,v_{m},$$

where each ($v_{i}$) denotes the coded value assigned to one taxonomy attribute based on the dataset paper, official documentation, repository, or access page. The profile (ϕ(D)) provides a compact representation of dataset variability, enabling datasets to be compared not only by application domain or sensing modality but also by the specific covariates that are represented, controlled, omitted, or insufficiently documented.

Each coded value ($v_{i}$) may correspond to one of four reporting states: a covariate is coded as *explicitly varied* when the documentation indicates that the factor changes across subjects, sequences, sessions, views, or acquisition conditions; it is coded as *controlled* when the factor is explicitly held constant by the acquisition protocol, such as a fixed indoor setting, a static background, or a single camera viewpoint; it is coded as *not reported* when the available documentation does not support reliable coding; finally, it is coded as *not applicable* when the attribute is structurally irrelevant to the dataset design. This distinction avoids treating missing documentation as evidence of absence and supports a conservative interpretation of covariate coverage.

The formal separation into three mutually exclusive subsets is governed by the following design principles, which ensure that the taxonomy remains analytically tractable and consistently applicable across heterogeneous datasets.

### 4.3. Design Properties of the Taxonomy

The taxonomy adheres to the following guiding principles:

- **Bounded coverage:** The taxonomy retains dominant and reproducibly documentable sources of variability, avoiding excessive fragmentation into dataset-specific or weakly reported attributes.
- **Origin-based coding:** Each covariate is assigned according to its primary source of variability in the acquisition and observation process. Environmental and contextual conditions are encoded as scene-level attributes, participant-related factors as user-level attributes, and acquisition or signal-formation properties as sensor-level attributes.
- **Non-redundancy:** No covariate is intentionally duplicated across dimensions. When a factor may affect multiple aspects of the observed gait signal, it is coded according to its primary origin rather than all downstream effects.
- **Controlled granularity:** Descriptors support both coarse comparison and refined specification when documentation allows. For example, a dataset may be described broadly as multi-view while also specifying the number, angular distribution, or height of the views when reported.
- **Modality-agnostic applicability:** The taxonomy is designed to apply across RGB, depth, RGB-D, silhouette, infrared, skeleton, pose, parsing, point-cloud, and multimodal gait datasets. Covariates are therefore defined at the level of dataset structure and acquisition variability rather than at the level of a single representation.
- **Protocol awareness:** The selected attributes map directly to common evaluation paradigms, including cross-view, cross-clothes, cross-speed, cross-time, cross-scene, cross-carrying, and cross-sensor evaluation. This supports a clearer connection between dataset design and benchmark interpretation.
- **Conservative coding:** Covariate values are assigned only when supported by dataset papers, official documentation, repositories, or access pages. Missing or ambiguous information is coded as *not reported* rather than inferred from unstated assumptions.

Together, these properties yield a compact yet expressive schema that links acquisition design, covariate structure, and evaluation methodology. The taxonomy therefore functions as an intermediary analytical layer between dataset documentation and algorithmic comparison, enabling consistent structural characterization across heterogeneous gait collections. In practical terms, it supports dataset selection, benchmark design, the identification of missing or under-reported covariates, and the interpretation of whether reported performance reflects robustness to specific sources of variability or performance under narrowly controlled acquisition conditions.

### 4.4. Scene-Level Covariates ($S_{\mathrm{scene}}$)

Scene-level covariates describe the environmental, spatial, geometric, and temporal conditions under which gait is observed. These attributes capture the variability introduced by the acquisition context rather than by the participant or by the sensing device itself. In the proposed taxonomy, scene-level covariates include the acquisition environment, background and scene complexity, walking surface or terrain, environmental control, viewpoint configuration, walking path, and temporal acquisition structure:

- **(A) Acquisition environment** identifies the general physical context in which data collection takes place. The values observed across the reviewed datasets range from controlled indoor environments, such as laboratories, corridors, clinical rooms, studios, gyms, museums, and stair setups, to outdoor environments, multi-scene outdoor settings, and in-the-wild locations. Some datasets combine indoor and outdoor acquisition. This attribute is intended to capture the broad environmental setting, while more specific aspects such as illumination control, background complexity, and camera arrangement are encoded separately.
- **(B) Background and scene complexity** describes the visual structure of the scene behind and around the walking subject. Common values include static backgrounds, green chroma-key backgrounds, real-world or varied backgrounds, dynamic backgrounds, cluttered scenes, crowded scenes, static obstacles, and scene-induced occlusion. This attribute is particularly relevant because background complexity and occlusion directly affect silhouette extraction, segmentation, pose estimation, and parsing-based representations.
- **(C) Walking surface or terrain** captures the physical support on which gait is performed. Most datasets use planar overground walking, but several introduce treadmill-constrained walking, treadmill incline, stairs, ramps, bumpy or soft surfaces, curved roads, or mixed terrain. This attribute is distinct from gait condition: the surface defines the physical constraint imposed by the environment, whereas walking style, pathology, speed, or activity condition are treated as user-level or protocol-related locomotion variables.
- **(D) Environmental control** specifies whether the acquisition conditions are controlled or uncontrolled. Controlled settings include laboratory, clinical, studio, or corridor-based acquisitions with stable lighting and constrained backgrounds. Uncontrolled settings include natural illumination, illumination changes, night-time acquisition, day/night variation, real-world outdoor conditions, or mixed indoor/outdoor protocols. This attribute provides a compact indication of environmental predictability and is useful for distinguishing laboratory-style benchmarks from more realistic surveillance or in-the-wild datasets.
- **(E) Viewpoint configuration** describes the perspective from which gait is observed. Values include single-view setups, single side-view acquisition, frontal-view acquisition, elevated side views, limited multi-view configurations, full 360-degree protocols, and large multi-camera or surveillance-style view distributions. Although viewpoint depends on camera placement, it is treated here as a scene-level covariate because it describes the geometric relation between the walking subject and the observation scene. The physical hardware arrangement itself, including camera type, number, placement, height, calibration, and synchronization, is encoded separately under sensor-level covariates.
- **(F) Walking path or trajectory** characterizes the spatial form of the walking route. Common values include straight trajectories, straight bidirectional walking paths, treadmill-constrained walking, curved trajectories, circular routes, figure-eight trajectories, square walking routes, cross-scene routes, round-trip paths with turns, assisted walking paths, mixed-task routes, and unconstrained trajectories. This attribute is important because trajectory structure affects body orientation, view transitions, occlusion patterns, and the temporal continuity of gait cycles.
- **(G) Temporal acquisition structure** captures how data collection is organized over time. Many datasets are single-session collections, whereas others involve multi-session acquisition, time gaps between recordings, long-run exhibition-based collection, seasonal variation, day/night variation, or collection periods spanning several months. When the temporal organization is not documented, the attribute should be coded as *not reported*. This covariate is particularly relevant for evaluating robustness to temporal change, re-acquisition effects, clothing seasonality, and long-term variability.

Together, these scene-level covariates provide a structured description of the acquisition context in which gait is captured. They allow datasets to be compared not only by domain or modality but also by the environmental realism, geometric complexity, route structure, and temporal variability represented in their acquisition protocols.

### 4.5. User-Level Covariates ($S_{\mathrm{user}}$)

User-level covariates describe variability associated with the participant being observed. These attributes capture factors related to locomotion, pace, appearance, carried objects, upper-limb configuration, and subject-level metadata. In the proposed taxonomy, user-level covariates include activity or gait condition, speed or pace condition, clothing or appearance condition, carrying/load and upper-limb condition, and participant descriptors.

- **(H) Activity or gait condition** identifies the locomotor task, walking pattern, or clinically relevant gait state represented in the dataset. The most common condition across the reviewed datasets is ordinary walking, but several datasets include additional activity or gait variants, such as running, stair ascent, speed-transition walking, stop/non-stop walking, prosthetic or pathological walking, simulated abnormal gait, asymmetric gait, Parkinsonian gait, knee osteoarthritis, scoliosis screening classes, freezing, limp, rigidity, or attribute-defined gait patterns. This attribute captures what the participant is doing or how gait is performed. It is distinct from scene-level terrain: for example, stairs as a physical surface are encoded as a scene-level covariate, whereas stair ascent as a locomotor task is encoded as a user-level gait condition.
- **(I) Speed or pace condition** describes how walking speed is determined, constrained, or varied. Common values include self-selected pace, slow walking, fast walking, speed-controlled walking, acceleration or deceleration protocols, stationary conditions, and treadmill-based constant-speed acquisition. Some datasets report explicit speed ranges or treadmill speeds, whereas others only indicate whether participants walked naturally or under imposed pace constraints. When speed is not described with sufficient detail, the attribute should be coded as *not reported*. This covariate is important because pace affects cadence, stride length, silhouette dynamics, pose trajectories, and temporal gait representations.
- **(J) Clothing or appearance condition** captures worn appearance factors that may modify the visible body shape or affect the extracted gait representation. Values observed across the reviewed datasets include natural clothing, explicit clothing variation, whole-body or partial clothing changes, coat or jacket conditions, lab coats, down jackets, footwear variation, shoes condition, and hat condition. This covariate is especially relevant for silhouette-, parsing-, and appearance-based recognition, since clothing can alter body contour, limb visibility, and segmentation quality. Clothing and worn accessories should be distinguished from external objects carried by the participant.
- **(K) Carrying/load and upper-limb condition** describes external objects carried by the participant, load-related conditions, or configurations that affect arm motion and body-part visibility. Common values include no load, bag, backpack, shoulder bag, handbag, travelling bag, luggage, book, box, heavy box, trolley, umbrella, ball, small or large carried items, object carrying, phone use, lift-stuff conditions, and hands-in-pockets. Although not all of these conditions correspond to physical load, they are grouped together because they can modify natural arm swing, occlude body regions, change silhouette shape, or introduce asymmetric motion. This attribute therefore supports cross-carrying and upper-limb-occlusion analysis.
- **(L) Participant descriptors** capture demographic, anthropometric, clinical, or contextual metadata associated with the subjects. Values observed across the reviewed datasets include age, sex, height, weight, body mass, ethnicity, nationality, profession, age group, physical activity level, and clinical severity. These descriptors do not always play the same role across tasks: they may function as target labels in attribute-recognition or healthcare-oriented datasets, as descriptive metadata in biometric datasets, or as covariates for stratified robustness and fairness analysis. When such information is unavailable or insufficiently specified, the attribute should be coded as *not reported*.

Together, these user-level covariates characterize how participant behavior, pace, appearance, carried objects, upper-limb configuration, and metadata shape the observable gait signal. They are essential for determining whether a dataset supports evaluation across walking styles, speeds, clothing conditions, carrying conditions, demographic groups, or clinically relevant populations.

### 4.6. Sensor-Level Covariates ($S_{\mathrm{sensor}}$)

Sensor-level covariates describe variability introduced by the sensing device, signal acquisition process, data representation, temporal and spatial sampling, synchronization strategy, and documented sensor-dependent acquisition limitations. These attributes capture how gait is recorded and represented, complementing the scene-level description of the acquisition context and the user-level description of participant-related variability. In the proposed taxonomy, sensor-level covariates include data modality or representation, frame rate or temporal sampling, spatial resolution, acquisition setup or sensor placement, synchronization or alignment, and sensor-dependent acquisition limitations.

- **(M) Data modality or representation** identifies the sensor data and derived representations made available by each dataset. Values observed across the reviewed datasets include RGB video, silhouettes, depth maps, infrared images, gait templates, 2D and 3D keypoints, pose representations, parsing maps, optical flow, point clouds, 3D volumes, 3D meshes, and gait descriptors. This attribute is intentionally broader than sensor type: it captures not only the raw acquisition modality but also the representations distributed or used as part of the dataset. This distinction is important because many gait benchmarks are evaluated on derived representations, such as silhouettes or GEI, rather than on raw video.
- **(N) Frame rate or temporal sampling** describes the temporal frequency at which gait data are acquired or represented. Common values include 10, 15, 20, 25, 30, 50, and 60 fps, although some datasets report mixed temporal rates across modalities, such as RGB and LiDAR streams, or include simulated low-frame-rate variants. When the acquisition or representation rate is not specified, the attribute should be coded as *not reported*. This covariate is particularly relevant because temporal sampling affects gait-cycle reconstruction, cadence estimation, event detection, motion smoothness, and the comparability of sequence-based models.
- **(O) Spatial resolution** captures the spatial size of the available visual or derived data. This may refer to RGB frames, depth maps, infrared images, silhouettes, GEI templates, optical-flow maps, parsing outputs, or other representations, depending on what is distributed with the dataset. Some datasets report a single image resolution, whereas others report different resolutions for different modalities or representations. When resolution is unavailable or only partially documented, the attribute should be coded conservatively as *not reported*. This covariate is important because spatial resolution influences segmentation quality, body-shape detail, keypoint localization, and the level of anatomical information preserved in the representation.
- **(P) Acquisition setup or sensor placement** describes the physical sensing arrangement used to capture gait. Values include fixed single-camera setups, multi-camera arcs, circular camera configurations, surveillance camera networks, Kinect-based RGB-D setups, thermal cameras, ASUS Xtion sensors, ceiling cameras, mobile robot-mounted RGB–LiDAR systems, and cameras placed at specified heights, distances, or angular layouts. This attribute differs from viewpoint configuration: viewpoint describes the observational perspective available in the dataset, whereas acquisition setup describes the physical hardware arrangement that produces those views.
- **(Q) Synchronization or alignment** specifies whether multiple streams, sensors, or views are temporally or geometrically coordinated. Values observed across the reviewed datasets include synchronized and calibrated multi-camera setups, timestamped multimodal streams, RGB–depth calibration or alignment, camera synchronization information, GPS-clock synchronization, external reference systems, not synchronized, not applicable, and not reported. This distinction is especially important for multi-view, multimodal, RGB-D, depth, skeleton, LiDAR, and reference-instrument datasets, where temporal or spatial misalignment can affect reconstruction, feature extraction, and cross-modal evaluation. For single-camera datasets without multiple streams, the attribute may be coded as *not applicable*.
- **(R) Sensor-dependent acquisition limitations** records limitations or artefacts that are directly attributable to the sensing device, recording hardware, or acquisition stream, as explicitly reported in the dataset documentation. This attribute is restricted to sensor-dependent issues such as frame loss, dropped frames, temporal gaps in the recorded stream, sensor noise, depth noise, LiDAR measurement noise, infrared/thermal sensor limitations, motion blur, rolling-shutter effects, sensor saturation, limited sensing range, or compression artefacts introduced at acquisition or recording time. This attribute should not be interpreted as a general quality score; it records only documented acquisition-level limitations that may affect reproducibility, temporal continuity, or signal reliability.

Together, these sensor-level covariates characterize how gait is encoded as data. They make explicit the acquisition and representation conditions that affect comparability across datasets, including modality, temporal sampling, spatial resolution, hardware configuration, synchronization, and known artefacts. These attributes are therefore essential for interpreting whether performance differences arise from gait-related variability, dataset design, sensing modality, representation quality, or preprocessing assumptions.

### 4.7. Boundary Cases and Coding Rules

Some covariates may influence multiple stages of the observation pipeline. To preserve consistent origin-based coding, each attribute is assigned according to its primary source of variability rather than according to all possible downstream effects. This principle is particularly important for borderline cases in which scene, user, and sensor factors interact.

The distinction between *walking surface or terrain* (C) and *activity or gait condition* (H) follows the same rule. Stairs, treadmill, ramp, or planar floor conditions are encoded as scene-level covariates when they describe the physical support or terrain. By contrast, stair ascent, treadmill walking, running, pathological walking, or simulated impaired gait are encoded as user-level covariates when they describe the locomotor task or gait condition performed by the participant.

A further boundary case concerns *viewpoint configuration* (E) and *acquisition setup or sensor placement* (P). Viewpoint configuration describes the effective observational perspective of the subject, such as side-view, frontal-view, multi-view, or 360-degree acquisition. Sensor placement describes the physical acquisition setup, including camera type, number, height, distance, tilt, mounting configuration, or sensor layout. Consequently, two datasets may share the same viewpoint label while differing substantially in physical sensor geometry.

Finally, background complexity, occlusion, and representation-quality issues should be interpreted according to their reported source. Static background, dynamic background, crowd presence, static obstacles, and scene-induced occlusion are encoded as scene-level properties when they describe the acquisition environment. Visibility changes caused by carried objects or upper-limb configuration are encoded under carrying/load and upper-limb condition (K). By contrast, sensor-dependent acquisition limitations (R) should be restricted to limitations directly attributable to the sensing device, recording hardware, or acquisition stream, such as frame loss, temporal gaps, sensor noise, depth noise, LiDAR noise, thermal/infrared acquisition limitations, motion blur, rolling-shutter effects, sensor saturation, limited sensing range, or acquisition-level compression. Silhouette extraction errors, pose-estimation failures, parsing errors, segmentation artefacts, and manual cropping should not be coded under R unless explicitly linked to sensor-level acquisition constraints.

### 4.8. Covariate Overview

Table 2 summarizes the proposed covariate taxonomy. The attributes are organized into scene-level, user-level, and sensor-level dimensions according to their primary source of variability. Each attribute may be explicitly varied, controlled, not reported, or not applicable, depending on the dataset documentation and acquisition protocol.

## 5. Taxonomy-Aligned Dataset Tables

This section operationalizes the proposed taxonomy by applying the A–R schema consistently to all 47 datasets included in the review. The results are presented in compact, modality-agnostic tables, with the datasets grouped according to their main application domain to improve readability and facilitate domain-specific comparisons. Within each table, the *scene-level* column encodes covariates A–G, the *user-level* column encodes covariates H–L, and the *sensor-level* column encodes covariates M–R. Detailed structural characterizations based on the normalized covariate values are provided in Table 3 for healthcare-oriented datasets, Table 4 for biometric-oriented datasets, and Table 5 for attribute-recognition datasets.

Appendix A complements these taxonomy-aligned tables by providing the complete list of the 47 reviewed datasets, grouped by main application domain and accompanied by the corresponding access information. Specifically, Table A1 lists the healthcare-oriented datasets, Table A2 lists the biometric-oriented datasets, and Table A3 lists the attribute-recognition datasets. Repository links and other available access routes are included to support the reproducibility of the review and were verified during manuscript preparation. Since dataset repositories, institutional webpages, and access procedures may change over time, this information should be understood as reflecting the access conditions available at the time of writing.

## 6. Analytical Insights Enabled by the Taxonomy

The proposed taxonomy enables an explicit and reproducible analysis of covariate coverage across heterogeneous gait datasets. By structuring variability along scene-level, user-level, and sensor-level dimensions, it becomes possible to identify methodological patterns that are often only described qualitatively or dispersed across dataset descriptions. The observations presented in this section are derived from the datasets instantiated in the taxonomy-aligned tables, covering healthcare-oriented, biometric-oriented, and attribute-recognition domains. Rather than exhaustively reporting all measurable covariates, the analysis focuses on those that reveal critical structural properties of the dataset landscape and expose limitations affecting generalization, comparability, and reproducibility.

### 6.1. Scene-Level Patterns

The dataset landscape remains strongly biased towards indoor and controlled acquisition. Of the 47 datasets reviewed, 36 (76.6%) were acquired exclusively indoors, 8 (17.0%) exclusively outdoors, and only 3 (6.4%) explicitly combine indoor and outdoor settings. This imbalance reflects the historical emphasis on reproducible acquisition protocols, particularly in laboratory, clinical, corridor, or exhibition-based environments. A similar pattern is observed for environmental control: 34 datasets (72.3%) were acquired under controlled conditions, 12 (25.5%) under uncontrolled conditions, and only 1 dataset (2.1%) combines controlled and uncontrolled acquisition scenarios. This confirms that ecological variability remains unevenly represented across the dataset landscape.

Background, surface, and trajectory covariates further reinforce this pattern. Most datasets rely on static backgrounds, including laboratory walls, corridors, green chroma-key setups, or other controlled visual environments, whereas dynamic backgrounds, scene-induced occlusion, static obstacles, multiple people, crowd effects, or staff presence appear mainly in more recent biometric or in-the-wild datasets. Similarly, planar overground walking and straight or straight bidirectional trajectories remain dominant, while treadmill walking, stairs, ramps, inclined treadmill conditions, bumpy or soft surfaces, curved routes, figure-eight paths, square routes, cross-scene trajectories, round-trip paths, mixed-task routes, or unconstrained trajectories are less frequent. This concentration on visually and geometrically simplified acquisition supports reproducibility and gait-cycle extraction, but limits evaluation under route, terrain, background, and orientation changes that are more common in real-world mobility.

Multi-view acquisition, captured by the viewpoint configuration covariate (E), is present in 27 of the 47 datasets (57.4%) but remains unevenly distributed across domains. Healthcare-oriented datasets remain mostly single-view, with only 3 of 11 datasets (27.3%) including multi-view acquisition. In contrast, 22 of the 33 biometric-oriented datasets (66.7%) and 2 of the 3 attribute-recognition datasets (66.7%) include multi-view configurations. This asymmetry is methodologically significant because viewpoint variability is central to evaluating cross-view generalization. Biometric datasets more frequently include structured multi-camera configurations, while healthcare-oriented datasets remain more clinically constrained and usually prioritize controlled observation over viewpoint diversity.

Temporal acquisition structure (G) remains one of the least consistently represented scene-level dimensions. Most datasets are single-session collections, accounting for 35 of the 47 datasets (74.5%). Eleven datasets (23.4%) report some form of extended temporal structure, such as long-run collection, collection periods spanning several months, exhibition-based acquisition, seasonal variation, time gaps, multi-session acquisition, or day/night acquisition. However, these cases should not all be interpreted as longitudinal protocols: reporting a collection period does not necessarily imply repeated acquisition of the same participants across time. A stricter reading shows that explicit multi-session, time-gap, day/night, or seasonal evaluation remains limited. This restricts the study of temporal drift, cross-session robustness, re-acquisition effects, and long-term model stability.

Overall, scene-level covariates reveal a dataset landscape still dominated by controlled indoor acquisition, static backgrounds, planar surfaces, straight trajectories, and single-session protocols. Recent biometric and attribute-recognition datasets increasingly introduce multi-view geometry, outdoor scenes, dynamic backgrounds, occlusion, and larger-scale acquisition, but these sources of scene variability remain unevenly distributed across domains. This imbalance has direct implications for benchmark design since model performance measured under controlled scene conditions may not translate to unconstrained, longitudinal, or environmentally complex gait-analysis scenarios.

### 6.2. User-Level Patterns

User-level variability exhibits a clear domain-dependent structure. Healthcare-oriented datasets predominantly emphasize locomotion condition (H), particularly pathological, prosthetic, clinically relevant, or simulated abnormal walking: 10 of the 11 healthcare datasets (90.9%) explicitly include pathological, clinical, or simulated abnormal gait conditions. In contrast, biometric-oriented datasets are primarily centered on identity recognition under normal walking, with robustness typically induced through variations in viewpoint, appearance, carrying condition, speed, or acquisition context. Attribute-recognition datasets occupy an intermediate position since they are not primarily clinical but explicitly encode semantic user-level labels such as age, sex, gait attributes, clothing, or body-related descriptors. Speed or pace condition (I) is less systematically varied than locomotion condition: only 12 of the 47 datasets (25.5%) explicitly include speed variation or speed-controlled acquisition, indicating that many benchmarks still evaluate gait under relatively narrow pace conditions.

Appearance-related covariates, particularly clothing configuration (J) and carrying/load or upper-limb condition (K), are more informative when interpreted as indicators of robustness-oriented dataset design rather than as requirements that should apply equally to all domains. Clothing or appearance variation is present in 18 of the 47 datasets (38.3%), including clothing changes, coat or jacket conditions, footwear variation, shoes condition, and hat condition. Carrying/load or upper-limb variation appears in 20 datasets (42.6%), including bags, backpacks, umbrellas, boxes, trolleys, carried items, phone use, and related conditions. These covariates are concentrated mainly in biometric datasets, where changes in body outline, occlusion, arm swing, and carried objects directly affect silhouette-based and appearance-based recognition.

Participant descriptors (L) are partially available but remain inconsistently reported. At least one participant-level descriptor is provided in 30 of the 47 datasets (63.8%), including 8 of the 11 healthcare datasets (72.7%), 19 of the 33 biometric datasets (57.6%), and all 3 attribute-recognition datasets. These descriptors include age, sex, height, weight or body mass, ethnicity, nationality, profession, age group, physical activity level, and clinical severity. However, the presence of basic descriptors such as age or sex does not necessarily imply comprehensive population characterization. Many datasets still provide limited information about demographic balance, age distribution, anthropometric variability, clinical stratification, or fairness-relevant user-level factors.

Overall, user-level covariates show that healthcare datasets prioritize clinically meaningful gait conditions, biometric datasets prioritize robustness to identity-preserving appearance and carrying variation, and attribute-recognition datasets prioritize semantic user labels. However, explicit speed variation and comprehensive participant metadata remain unevenly represented across the dataset landscape.

### 6.3. Sensor-Level Patterns

At the sensor level, variability is strongly shaped by the dominance of silhouette-centered representations (M). When direct silhouettes and derived silhouette-based representations such as GEI and SEI are treated jointly, this family is present in 39 of the 47 datasets reviewed (83.0%). Direct silhouettes (SIL) are reported in 38 datasets (80.9%) and GEI in 10 datasets (21.3%). By contrast, explicit depth data (DPT) appear in only 6 datasets (12.8%), infrared data (IR) in 3 datasets (6.4%), and RGB data in 17 datasets (36.2%). Keypoint-based representations are also present, with 2D keypoints reported in 14 datasets (29.8%) and 3D keypoints in 9 datasets (19.1%). This distribution shows that, despite the increasing availability of RGB-D sensors, pose-estimation pipelines, and multimodal acquisition setups, the reviewed dataset landscape remains strongly centered on 2D silhouette-based gait representations.

Temporal and spatial sampling remain heterogeneous. Frame rates range from 10 fps to 60 fps, with 25 fps and 30 fps appearing frequently, while some datasets include mixed temporal rates across modalities or simulated low-frame-rate variants. Spatial resolution also varies substantially, from low-resolution silhouettes or gait templates such as 64 × 44 and 128 × 88 to high-resolution RGB or silhouette frames such as 1920 × 1080. This variation is methodologically relevant because temporal sampling and spatial resolution directly affect gait-cycle reconstruction, event detection, keypoint localization, silhouette quality, and the comparability of sequence-based models.

Synchronization and alignment (Q) should be interpreted broadly since they are relevant not only to multi-camera datasets but also to multimodal, RGB-D, LiDAR, external-reference, and timestamped acquisition setups. Excluding datasets for which synchronization is not applicable, 29 datasets require some form of synchronization, alignment, or timing information. Among these, 19 datasets (65.5%) explicitly report synchronized acquisition, timestamped multimodal streams, RGB–depth alignment, GPS-clock synchronization, camera synchronization information, or external reference timing. One dataset (3.4%) explicitly reports non-synchronized acquisition, while 9 datasets (31.0%) do not report synchronization or alignment status. This distinction is important because multi-view, multimodal, or reference-based acquisition does not automatically imply temporally or geometrically aligned data.

Overall, sensor-level covariates reveal a dataset landscape dominated by silhouette-based representations, heterogeneous temporal and spatial sampling, and incomplete reporting of synchronization or alignment. Sensor-dependent acquisition limitations (R) are also rarely documented in a structured way: under the revised definition, only frame loss, LiDAR noise, and acquisition-level compression can be interpreted as direct sensor- or recording-dependent limitations, while temporal subsampling and spatial downscaling are better treated as representation or protocol-level transformations unless explicitly linked to the acquisition hardware. This underreporting may affect reproducibility, signal reliability, and cross-dataset comparability.

### 6.4. Covariate Value Distributions

Binary covariate coverage is useful for determining whether a taxonomy attribute is represented, but it is often insufficient to characterize how datasets differ in practice. For several attributes, the most informative level of analysis lies not in the mere presence of the covariate, but in the distribution of its normalized coding values. For example, reporting that a dataset includes walking-surface information is less informative than distinguishing between overground, treadmill, stairs, or mixed-terrain acquisition. Similarly, viewpoint configuration is more meaningful when separated into single-view and multi-view settings, and synchronization is more informative when distinguished as reported, not applicable, explicitly absent, or not reported.

**Figure 3:**
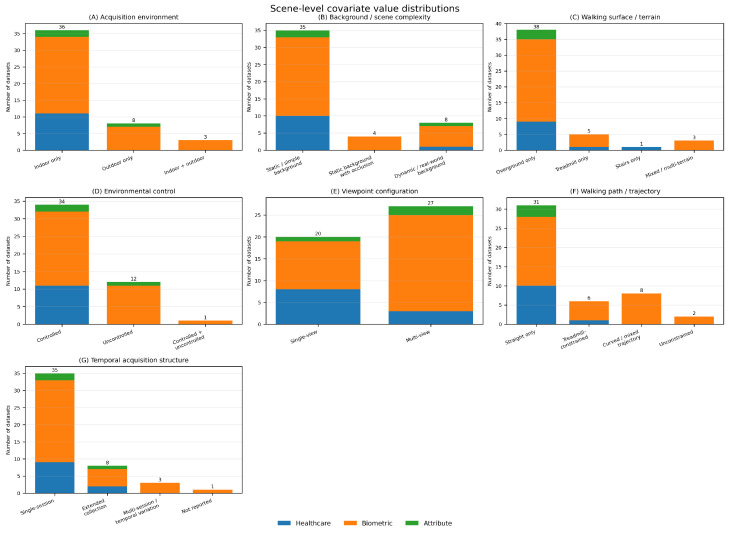
Scene-level covariate value distributions across the 47 reviewed datasets, stratified by application domain. The figure summarizes normalized coding values for acquisition environment (**A**), background and scene complexity (**B**), walking surface or terrain (**C**), environmental control (**D**), viewpoint configuration (**E**), walking path or trajectory (**F**), and temporal acquisition structure (**G**).

**Figure 4:**
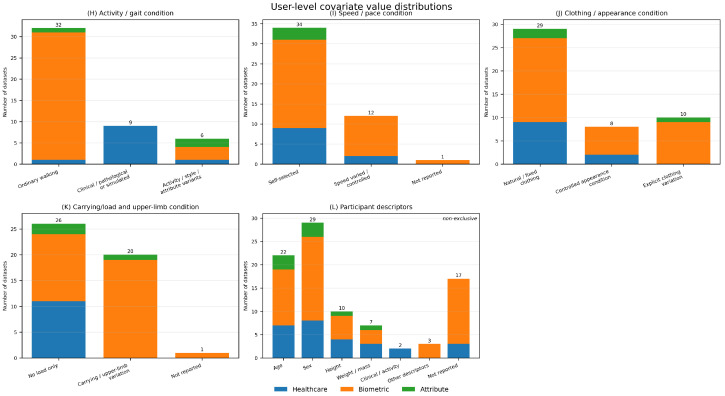
User-level covariate value distributions across the 47 reviewed datasets, stratified by application domain. The figure summarizes the normalized coding values for a selection of user-level covariates: activity or gait condition (**H**), speed or pace condition (**I**), clothing or appearance condition (**J**), carrying/load condition (**K**), and participant descriptors (**L**). Participant descriptor categories are non-exclusive, as a single dataset may report multiple descriptor types.

**Figure 5:**
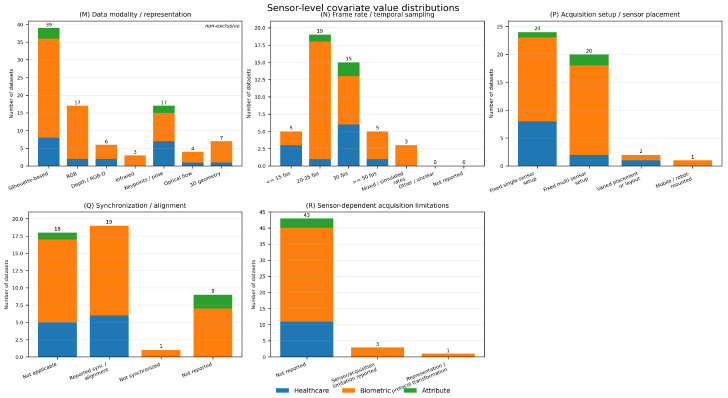
Sensor-level covariate value distributions across the 47 reviewed datasets, stratified by application domain. The figure summarizes the normalized coding values for selected sensor-level covariates: data modality or representation (**M**), frame rate or temporal sampling (**N**), acquisition setup or sensor placement (**P**), synchronization or alignment (**Q**), and sensor-dependent acquisition limitations (**R**). Categories in the modality panel are non-exclusive, as a single dataset may include multiple sensing modalities or derived representations.

For this reason, the analysis was extended from attribute-level coverage to value-level distributions for selected covariates whose internal categories directly affect benchmark interpretation, robustness claims, and cross-dataset comparability. Figure 3, Figure 4, and Figure 5 summarize these distributions for scene-level, user-level, and sensor-level attributes, respectively. The selected attributes were chosen because their values can be normalized into interpretable categories with direct methodological relevance. Attributes whose values are highly heterogeneous, dataset specific, or primarily descriptive rather than distributional are better interpreted through the taxonomy-aligned tables rather than through compact summary plots.

At the scene level, Figure 3 shows that the reviewed datasets remain strongly concentrated in controlled and simplified acquisition settings. Of the 47 datasets, 36 are indoor-only, 8 are outdoor-only, and only 3 explicitly combine indoor and outdoor acquisition. Background conditions are similarly concentrated: 35 datasets use static or simple backgrounds, whereas 4 include static backgrounds with occlusion and 8 include dynamic or real-world backgrounds. Walking surface is dominated by overground acquisition, with 38 overground-only datasets, compared with 5 treadmill-only datasets, 1 stairs-only dataset, and 3 mixed- or multi-terrain datasets. Environmental control follows the same pattern, with 34 controlled datasets, 12 uncontrolled datasets, and only 1 dataset combining controlled and uncontrolled conditions.

Viewpoint and trajectory provide a more nuanced picture. Multi-view acquisition is present in 27 datasets, while 20 are single-view. However, multi-view acquisition is concentrated mainly in biometric and attribute-recognition datasets, whereas healthcare-oriented datasets remain predominantly single-view. Walking trajectories are also largely simplified: 31 datasets use straight-only routes, 6 are treadmill-constrained, 8 include curved or mixed trajectories, and only 2 involve unconstrained trajectories. Temporal acquisition structure remains particularly sparse, with 35 single-session datasets, 8 extended collection protocols, 3 explicit multi-session or temporal-variation protocols, and 1 dataset for which the temporal structure is not reported.

At the user level, Figure 4 highlights clear domain-dependent priorities. Ordinary walking dominates the dataset landscape, appearing as the main activity condition in 32 datasets. Clinical, pathological, or simulated abnormal gait conditions appear in 9 datasets, mainly in the healthcare-oriented domain, while activity-, style-, or attribute-based variants appear in 6 datasets. Speed is also weakly diversified: 34 datasets rely on self-selected walking, 12 include speed variation or speed-controlled acquisition, and 1 does not report speed information.

Appearance and carrying conditions are more strongly associated with biometric robustness evaluation. Natural or fixed clothing is used in 29 datasets, 8 datasets include controlled appearance conditions such as coats, jackets, footwear, or hats, and 10 datasets include explicit clothing variation. Carrying/load and upper-limb conditions show a similar distribution: 26 datasets include no-load walking only, 20 include carrying or upper-limb variation, and 1 does not report this information. Participant descriptors are shown as non-exclusive categories because a single dataset may report multiple descriptors. Sex is reported in 29 datasets and age in 22 datasets, whereas height, weight or body mass, clinical/activity descriptors, and other descriptors are less consistently available. Seventeen datasets do not report participant descriptors in the normalized coding table.

At the sensor level, Figure 5 shows that the dataset landscape remains strongly centered on silhouette-based representations. Because datasets may include multiple modalities or derived representations, the modality panel is non-exclusive. Silhouette-based representations are present in 39 datasets, while RGB and keypoint/pose representations each appear in 17 datasets. Depth or RGB-D data appear in 6 datasets, 3D geometric representations in 7, optical flow in 4, and infrared data in 3. This confirms that, despite the growth of multimodal and pose-based approaches, silhouette-based representations remain the dominant basis for image- and depth-based gait benchmarking.

Temporal sampling and acquisition setup are also unevenly distributed. Frame rates are concentrated around 20–25 fps and 30 fps, with 19 and 15 datasets respectively, while only 5 datasets use 15 fps or lower, 5 use 50 fps or higher, and 3 include mixed or simulated temporal rates. Acquisition setup is split between fixed single-sensor setups in 24 datasets and fixed multi-sensor setups in 20 datasets, with only 2 datasets involving varied placement or layout and 1 using a mobile or robot-mounted acquisition system. Synchronization and alignment are reported in 19 datasets, not applicable in 18, not reported in 9, and explicitly absent in 1. Finally, sensor-dependent acquisition limitations remain rarely documented: 43 datasets do not report such limitations, 3 report sensor- or acquisition-level limitations, and 1 includes a representation- or protocol-level transformation that should not be interpreted as a strictly sensor-dependent acquisition artefact.

Taken together, these value-level distributions provide a more concrete view of dataset structure than binary covariate presence alone. They show not only which covariates are represented, but also how their values are distributed across the current dataset landscape. This supports more precise interpretation of benchmark scope, robustness claims, domain-specific design priorities, and remaining structural gaps in publicly accessible image- and depth-based gait datasets.

### 6.5. Synthesis

Taken as a whole, these observations demonstrate that the proposed taxonomy enables a structured, reproducible, and task-aware analysis of heterogeneous gait datasets. Rather than treating datasets only as benchmark sources, the taxonomy makes it possible to examine how scene-level, user-level, and sensor-level covariates are represented, controlled, omitted, or insufficiently documented.

In particular, the analysis supports:

- Explicit quantification of covariate coverage across heterogeneous datasets;
- Identification of domain-specific biases in dataset design;
- separation of scene-level, user-level, and sensor-level sources of variability;
- Distinction between explicitly varied, controlled, not reported, and not applicable covariates;
- Identification of underrepresented dimensions, particularly temporal acquisition structure (G), synchronization or alignment (Q), and sensor-dependent acquisition limitations (R);
- Analysis of normalized covariate value distributions, enabling concrete comparison of how datasets differ within each taxonomy attribute.

These results support the claim that a covariate-centered, modality-agnostic representation is not only descriptive but analytically enabling. It provides a concrete basis for dataset comparison, protocol design, robustness evaluation, and more transparent reporting practices in gait analysis. At the same time, the interpretation of covariate coverage should remain task-conditional: not every dataset is expected to vary every covariate, but each dataset should clearly document which covariates are varied, controlled, not applicable, or not reported.

## 7. Structural Gaps and Research Implications

The taxonomy-driven analysis presented in the previous section reveals recurring structural patterns in current gait datasets. These patterns should not be interpreted as universal deficiencies since covariate coverage is necessarily shaped by the intended application, acquisition constraints, and evaluation objectives of each dataset. For example, healthcare-oriented datasets are often expected to rely on controlled indoor acquisition, stable illumination, static backgrounds, and fixed sensor placement because their primary goal is to isolate clinically relevant locomotor patterns rather than to challenge models with environmental variability. Conversely, biometric and attribute-recognition datasets are more often designed to assess robustness under viewpoint, appearance, and acquisition variability.

Accordingly, the implications discussed in this section are framed as *task-conditional structural gaps:* a covariate should be considered underrepresented only when its absence limits the stated or implied evaluation objective of a dataset or benchmark. By linking each issue to the corresponding covariate groups, the discussion translates descriptive coverage statistics into actionable implications for dataset design, documentation, and evaluation protocol definition.

### 7.1. Application-Dependent Ecological Diversity in Acquisition Settings (A, B, D)

The predominance of controlled indoor acquisition reflects different methodological priorities across application domains. In healthcare-oriented datasets, controlled environments, static backgrounds (B), and stable illumination conditions (D) are often appropriate and even desirable since they reduce confounding factors and support the analysis of pathological or clinically relevant gait patterns. In this context, the absence of outdoor acquisition should not be interpreted as a limitation by itself.

However, the same controlled acquisition strategy becomes limiting when datasets are used to support claims about environmental robustness, real-world deployment, or cross-context generalization. In biometric and surveillance-inspired settings, narrow ecological diversity may reduce external validity, particularly when models are expected to operate under changing backgrounds, illumination, surface conditions, or acquisition contexts.

**Research implication.** Dataset documentation should explicitly state whether environmental conditions are intentionally controlled, actively varied, or simply unreported. When robustness to environmental variability is claimed, datasets should include dedicated evaluation splits or scenarios reflecting variation in environment type (A), background structure (B), and illumination conditions (D). When the purpose is clinical isolation rather than ecological robustness, controlled acquisition should be reported as a deliberate design choice rather than treated as an omission.

### 7.2. Uneven Viewpoint Coverage Across Domains (E, P)

Viewpoint diversity is strongly domain-dependent. Healthcare datasets remain largely single-view, whereas biometric and attribute-recognition datasets more frequently provide multi-view acquisition. This asymmetry reflects different application priorities: clinical gait analysis often benefits from standardized and repeatable sensor placement, while biometric evaluation commonly requires robustness to viewpoint variation.

Thus, limited viewpoint diversity in healthcare datasets is not necessarily problematic when the evaluation objective is controlled clinical assessment. It becomes a limitation when such datasets are used to evaluate viewpoint-invariant methods, cross-view transfer, or deployment scenarios in which camera placement cannot be standardized. In this sense, the structural issue is not the prevalence of single-view healthcare datasets but the lack of explicit alignment between viewpoint coverage and the claims made by evaluation protocols.

**Research implication.** Cross-view protocols should report both the effective subject-to-camera viewpoint (E) and the physical sensor placement geometry (P), since similar viewpoint labels may correspond to different camera heights, distances, tilt angles, or mounting configurations. Future datasets should provide more diverse observation geometries when viewpoint robustness or cross-view transferability is a stated objective. Conversely, when fixed viewpoint acquisition is intentional, it should be documented as part of the protocol design.

### 7.3. Scarcity of Temporal Acquisition Structure and Longitudinal Protocols (G)

Temporal acquisition structure (G) remains one of the clearest underrepresented dimensions in the reviewed dataset landscape. Unlike controlled lighting or indoor acquisition, which may be justified in several domains, the lack of repeated acquisition across time has broader implications. Most datasets rely on single-session acquisition, and longitudinal or multi-session protocols remain largely restricted to biometric scenarios.

This is particularly relevant because temporal variation can affect both biometric and healthcare applications. In biometrics, it relates to the persistence of identity cues over time; in healthcare, it is central to monitoring disease progression, rehabilitation outcomes, or changes in functional mobility. Therefore, the scarcity of longitudinal structure limits the study of temporal drift, intra-subject evolution, session variability, and long-term model stability.

**Research implication.** When temporal robustness, subject monitoring, or longitudinal clinical interpretation is claimed, datasets should include repeated acquisitions across distinct sessions and report the time interval between sessions. Cross-time protocols should distinguish short-term repetition from long-term temporal variation since session gaps, changes in appearance, sensor repositioning, and environmental variation may affect model stability in different ways.

### 7.4. Domain-Specific Coverage of User-Related Covariates (H, J, K)

User-level covariates are unevenly represented across application domains, but this asymmetry is partly expected. Healthcare-oriented datasets strongly emphasize locomotion condition (H), particularly pathological or simulated abnormal gait, whereas biometric and attribute-recognition datasets place greater emphasis on appearance-related variation, including clothing configuration (J) and carrying/load and upper-limb condition (K). This reflects legitimate differences in dataset purpose.

The key issue is therefore not that healthcare datasets generally lack clothing or carrying variation but that user-related covariates are rarely modeled jointly across domains. For example, clinical datasets often isolate gait pathology under controlled appearance conditions, while biometric datasets often model appearance variation without clinical labels. This separation limits the study of interactions between functional gait differences and realistic visual variability.

**Research implication.** Protocols should distinguish appearance variation caused by clothing from silhouette or motion changes induced by carried objects, rather than merging these conditions into generic covariate labels. Where appropriate, future datasets should adopt combinatorial designs that jointly vary locomotion condition (H), clothing configuration (J), and carrying condition (K), especially when the intended application involves deployment outside controlled clinical or laboratory conditions.

### 7.5. Incomplete Demographic Characterization (L)

Demographic annotation (L) remains inconsistent across the reviewed datasets. Although some datasets report basic descriptors such as sex and age, richer demographic characterization is less common and unevenly distributed across application domains. Unlike some acquisition covariates, demographic reporting is broadly relevant across domains because it affects fairness-aware evaluation, population-level interpretation, and the assessment of model generalization across subject groups.

At the same time, demographic reporting is constrained by ethical, privacy, and regulatory considerations. Therefore, the issue is not that all datasets must provide complete individual-level demographic metadata, but that the availability, absence, or restriction of such metadata should be clearly documented.

**Research implication.** Demographic metadata should be reported at least at an aggregate level when ethically and legally appropriate, together with clear indication of missing, unavailable, or privacy-restricted fields. Dataset documentation should distinguish between uncollected demographic variables, variables collected but not released, and variables intentionally omitted for ethical reasons. This distinction is essential for fairness-aware evaluation and population-level interpretation.

### 7.6. Modality Concentration Around Silhouette-Based Representations (M)

The modality distribution remains strongly concentrated around silhouette-based data, including direct silhouettes and derived encodings such as GEI and SEI. This concentration is historically understandable since silhouettes are compact, privacy-preserving, and effective for many gait recognition pipelines. In large-scale biometric benchmarking, silhouette-based representations also support standardized comparison across methods.

However, modality concentration becomes limiting when the research objective involves clinical interpretability, geometry-aware analysis, multimodal fusion, or cross-modal transfer. In such cases, reliance on silhouette representations alone may discard information related to body shape, depth, texture, skeletal structure, or fine-grained movement dynamics.

**Research implication.** Dataset documentation should explicitly distinguish raw modalities from derived representations, for example RGB, depth, silhouettes, GEI, skeletons, keypoints, or parsing masks. Future benchmarks should clarify whether evaluation is intended to assess modality-specific performance, representation robustness, or cross-modal transfer since these objectives require different protocol designs and reporting standards.

### 7.7. Incomplete Reporting of Synchronization and Alignment (Q)

Synchronization and alignment are relevant not only for multi-camera datasets but also for multimodal, RGB-D, LiDAR, timestamped, and external-reference acquisition setups. This issue is particularly important because multi-view acquisition does not necessarily imply temporally synchronized data. A dataset may provide multiple views for independent recognition experiments without supporting temporally aligned multi-view fusion, reconstruction, or cross-view temporal correspondence.

The absence of synchronization reporting is therefore not always a flaw in the dataset itself, but it becomes a methodological limitation when studies assume temporal alignment or use the data for tasks requiring synchronized streams.

**Research implication.** Multi-camera datasets should explicitly state whether streams are synchronized, independently recorded, or temporally unaligned, and should avoid treating multi-view acquisition as equivalent to synchronized acquisition. When synchronization is present, the mechanism should be reported; when it is absent, protocols should avoid assuming temporal correspondence across views.

### 7.8. Underreported Sensor-Dependent Acquisition Limitations (R)

Sensor-dependent acquisition limitations (R) remain rarely documented as structured dataset properties. Under the proposed definition, this attribute is restricted to limitations directly attributable to the sensing device, recording hardware, or acquisition stream, such as frame loss, dropped frames, temporal gaps in the recorded stream, sensor noise, depth noise, LiDAR measurement noise, infrared or thermal sensor limitations, motion blur, rolling-shutter effects, sensor saturation, limited sensing range, or compression artifacts introduced at acquisition or recording time.

This restricted interpretation is important because it avoids conflating acquisition-level limitations with downstream representation or preprocessing issues. For example, silhouette extraction errors, pose-estimation failures, parsing errors, segmentation artifacts, manual cropping, and background-subtraction dependence should not be coded under R unless they are explicitly caused by sensor-level acquisition constraints. Similarly, occlusion should be treated as a scene-level or user-level factor when it originates from the environment or carried objects, rather than as a sensor-dependent limitation.

**Research implication.** Dataset documentation should explicitly report known sensor-dependent acquisition limitations when they are present, and should distinguish them from preprocessing or representation-quality issues. This distinction is necessary for interpreting whether performance degradation arises from the sensing process itself, from the scene or participant configuration, or from downstream data processing.

### 7.9. Dataset Access, Annotation Traceability, and Documentation Quality

Beyond covariate coverage, dataset reusability also depends on access stability, licensing clarity, annotation traceability, and sufficient documentation of acquisition and evaluation protocols. Public availability alone does not guarantee reproducibility if access conditions are unclear, metadata are incomplete, annotation procedures are not described, or recommended evaluation splits are absent.

This issue is particularly relevant for heterogeneous gait datasets, where labels may refer to identities, clinical groups, demographic descriptors, attributes, clothing conditions, carrying conditions, pose representations, or derived silhouettes. Without clear documentation of how these annotations were obtained, whether they were manually verified, automatically estimated, or inherited from preprocessing pipelines, it becomes difficult to interpret benchmark results or compare methods across datasets.

**Research implication.** Future dataset releases should document not only the data modalities and covariates represented but also access conditions, licensing or data-use restrictions, annotation procedures, class or attribute balance, demographic composition where appropriate, and recommended evaluation protocols. When information cannot be released for ethical, legal, or privacy reasons, this should be stated explicitly.

### 7.10. Lack of Standardized Reporting Across Datasets

A broader structural issue cuts across all covariate groups: the absence of a consistent reporting standard. Importantly, standardized reporting does not imply that all datasets should vary all covariates. Rather, it requires that datasets clearly indicate which covariates are actively varied, which are intentionally controlled, which are not applicable, and which are not reported. This distinction is essential to avoid misinterpreting controlled design choices as omissions or treating missing metadata as evidence of absence.

**Research implication.** Future dataset documentation should clearly separate absent covariates, fixed or controlled covariates, actively varied covariates, not applicable fields, and unreported fields. The proposed taxonomy offers a practical basis for such standardized reporting, enabling dataset descriptions to directly support reproducibility, benchmark design, and covariate-aware evaluation without imposing the same variability requirements on all application domains.

### 7.11. Synthesis

Taken together, these structural patterns show that the current dataset ecosystem is not uniformly deficient, but unevenly aligned with different evaluation objectives. Controlled acquisition is appropriate for some purposes, particularly clinical isolation and pathology-focused analysis, whereas broader covariate coverage is essential for claims about robustness, generalization, and real-world deployment.

The main contribution of the proposed taxonomy is therefore not simply to expose missing variability but to clarify the status of each covariate as actively varied, controlled, not applicable, or not reported. This distinction supports task-appropriate dataset design and prevents controlled protocol choices from being misinterpreted as omissions.

The analysis also shows that dataset documentation remains as important as dataset scale. Future progress in gait analysis will depend not only on larger collections, but also on clearer reporting of acquisition conditions, participant descriptors, sensing configuration, synchronization or alignment, annotation procedures, access conditions, and known sensor-dependent acquisition limitations. This creates a more precise basis for designing future datasets, defining evaluation protocols, and supporting robust, fair, and interpretable gait-analysis research.

## 8. Conclusions

This work presented a systematic review of publicly accessible image- and depth-based gait datasets and introduced a covariate-centered structural taxonomy that separates scene-level, user-level, and sensor-level sources of variability. The taxonomy was operationalized through a compact A–R coding scheme and instantiated in taxonomy-aligned tables, enabling consistent comparison across heterogeneous datasets, application domains, and sensing modalities.

The analysis shows that current gait datasets exhibit substantial imbalances in covariate coverage and reporting practices. The reviewed dataset landscape remains strongly oriented toward controlled acquisition settings, static or constrained scenes, planar walking, straight trajectories, and single-session protocols. Temporal acquisition structure, comprehensive participant descriptors, synchronization or alignment information, and sensor-dependent acquisition limitations remain unevenly documented. These limitations affect cross-dataset comparability, robustness evaluation, and the interpretation of generalization claims in gait-analysis research.

Importantly, these findings should be interpreted in a task-conditional manner. The absence of a covariate is not necessarily a dataset flaw: controlled acquisition may be appropriate for clinical isolation, while broader environmental and appearance variability may be essential for biometric or surveillance-oriented robustness. The proposed taxonomy therefore does not impose the same variability requirements on all datasets. Instead, it provides a structured way to distinguish covariates that are actively varied, intentionally controlled, not applicable, or not reported.

The taxonomy also provides practical guidance for dataset reporting and benchmark design. Dataset documentation should explicitly describe acquisition environment, background structure, walking surface, environmental control, viewpoint configuration, trajectory structure, temporal acquisition structure, locomotion condition, walking speed, clothing and carrying/load conditions, participant descriptors, data modality or representation, frame rate, spatial resolution, sensor placement, synchronization or alignment, and known sensor-dependent acquisition limitations. When information is unavailable, restricted, or not applicable, this should be stated explicitly.

Future work should therefore move beyond the collection of larger datasets alone and focus on better documented, protocol-aligned, and covariate-aware dataset design. Standardized metadata schemas derived from the proposed taxonomy could support more transparent benchmarking, more reliable cross-dataset comparison, and more robust, fair, and generalizable gait-analysis systems in clinical, biometric, and real-world deployment contexts.

## Figures and Tables

**Table 1:** Positioning of the proposed taxonomy with respect to representative gait-analysis surveys and classification frameworks.

Work	Primary Organizing Principle	Relation to the Present Work
Sepas-Moghaddam and Etemad [2]	Body, temporal, feature, and architectural representations	Focuses on deep recognition models rather than dataset-level covariate coverage.
Shen et al. [3]	Deep representations, architectures, benchmarks, and challenges	Surveys deep gait recognition, treating datasets mainly as evaluation benchmarks.
Santos et al. [1]	Deep learning methods, datasets, architectures, and limitations	Emphasizes recognition pipelines and performance, not dataset variability or reporting consistency.
Nambiar et al. [4]	Gait-based person re-identification methods and evaluation	Focuses on re-identification rather than cross-domain dataset covariate characterization.
Parashar et al. [9]	Covariates and deep learning strategies for handling them	Addresses covariates mainly as robustness challenges for recognition algorithms.
Han et al. [5]	Vision sensors, machine learning methods, and applications	Reviews sensor systems and applications, not dataset-level covariate taxonomy.
Nunes et al. [6]	Depth/RGB-D gait dataset properties and availability	Reviews RGB-D gait datasets, while the present work covers broader modalities and domains.
**Present work**	**Scene-level, user-level, and sensor-level covariate coverage**	**Treats dataset variability as the primary object of analysis and provides a structured taxonomy for dataset characterization, comparison, documentation, and benchmark design.**

**Table 2:** Overview of the proposed covariate-centered taxonomy organized into scene-level, user-level, and sensor-level attributes.

Dimension	Code	Taxonomy Attribute
Scene-level	A–G	Acquisition environment; background and scene complexity; walking surface or terrain; environmental control; viewpoint configuration; walking path or trajectory; temporal acquisition structure.
User-level	H–L	Activity or gait condition; speed or pace condition; clothing or appearance condition; carrying/load condition; participant descriptors.
Sensor-level	M–R	Data modality or representation; frame rate or temporal sampling; spatial resolution; acquisition setup or sensor placement; synchronization or alignment; sensor-dependent acquisition limitations.

**Table 3:** **Healthcare-oriented gait datasets** organized under the proposed covariate taxonomy. Each dataset is characterized according to scene-level ($S_{\mathrm{scene}}$), user-level ($S_{\mathrm{user}}$), and sensor-level ($S_{\mathrm{sensor}}$) factors, following the structured covariate framework (A–R) (see Section 4).

Dataset	Year	Subjects	Scene-Level	User-Level	Sensor-Level
Gait-A Database [43]	2016	5 (73 samples: 38 normal; 35 abnormal)	**(A)** Indoor (corridor) **(B)** Static background **(C)** Planar (overground) **(D)** Controlled **(E)** Multi-view (2 views: side; front) **(F)** Straight trajectory **(G)** Single-session	**(H)** Walking (Normal; Simulated abnormal gait) **(I)** Self-selected **(J)** Natural clothing **(K)** No load **(L)** Not reported	**(M)** SIL **(N)** 30 fps **(O)** 1920 × 1080 **(P)** Fixed single camera **(Q)** Not applicable **(R)** Not reported
GAIT-IST [14]	2020	10 (8 ♂–2 ♀)	**(A)** Indoor (laboratory setting) **(B)** Static background **(C)** Planar (overground) **(D)** Controlled **(E)** Single side view **(F)** Straight bidirectional walking path **(G)** Single-session	**(H)** Walking (Normal; Diplegic; Hemiplegic; Neuropathic; Parkinsonian) **(I)** Self-selected **(J)** Natural clothing **(K)** No load **(L)** Age; Sex	**(M)** SIL; GEI; SEI; 2D-KPT (OpenPose) **(N)** 25 fps **(O)** 224 × 224 **(P)** Fixed single camera (1.5 m height; ≈4 m distance) **(Q)** Not applicable **(R)** Not reported
GAIT-IT [17]	2021	21 (19 ♂–2 ♀)	**(A)** Indoor (laboratory setting) **(B)** Static background (green chroma-key) **(C)** Planar (overground) **(D)** Controlled **(E)** Multi-view (2 views: side; front) **(F)** Straight bidirectional walking path **(G)** Single-session	**(H)** Walking (Normal; Scissor; Spastic; Steppage; Propulsive) **(I)** Self-selected **(J)** Natural clothing **(K)** No load **(L)** Age; Sex	**(M)** SIL; GEI; SEI **(N)** 10 fps **(O)** 224 × 224 **(P)** Fixed 2 cameras (1.75 m height) **(Q)** Synchronized calibrated multi-camera setup **(R)** Not reported
Health&Gait [19]	2025	398 (199 ♂–199 ♀)	**(A)** Indoor (laboratory setting) **(B)** Static background **(C)** Planar (overground) **(D)** Controlled **(E)** Single side view **(F)** Straight bidirectional walking path **(G)** Eight-month collection	**(H)** Walking **(I)** Self-selected; Fast **(J)** Natural clothing; Jacket condition **(K)** No load **(L)** Age; Sex; Height; Weight; Physical activity level	**(M)** SIL; 2D-KPT (AlphaPose); 2D-PRS; OF **(N)** 30 fps **(O)** SIL: 960 × 540; OF: 480 × 270 **(P)** Fixed single camera (placement varied by session) **(Q)** OptoGait/MuscleLAB reference data; DSU 1 ms for photocells **(R)** Not reported
INIT Gait Database [15]	2016	10 (9 ♂–1 ♀)	**(A)** Indoor (laboratory setting) **(B)** Static background (green chroma-key) **(C)** Planar (overground) **(D)** Controlled **(E)** Single side view **(F)** Straight trajectory **(G)** Single-session	**(H)** Walking (Normal; 7 simulated impaired gait styles) **(I)** Self-selected **(J)** Natural clothing **(K)** No load **(L)** Sex	**(M)** SIL **(N)** 15 fps **(O)** 800 × 400 **(P)** Fixed single camera **(Q)** Not applicable **(R)** Not reported
KOA-PD-NM [18]	2020	96 (50 KOA; 16 PD; 30 NM)	**(A)** Indoor (clinical setting) **(B)** Static background (green chroma-key) **(C)** Planar (overground) **(D)** Controlled **(E)** Single side view **(F)** Straight bidirectional walking path **(G)** 2018–2019 collection	**(H)** Walking (Normal; Knee Osteoarthritis; Parkinsonian) **(I)** Self-selected **(J)** Natural clothing **(K)** No load **(L)** Age; Sex; Height; Clinical severity	**(M)** RGB **(N)** 50 fps **(O)** 1920 × 1080 **(P)** Fixed single camera (8 m from walking mat) **(Q)** Not applicable **(R)** Not reported
MMGS [44]	2019	27 (19 ♂–8 ♀)	**(A)** Indoor (laboratory setting) **(B)** Static background **(C)** Planar (overground) **(D)** Controlled **(E)** Single frontal view **(F)** Straight trajectory **(G)** Single-session	**(H)** Walking (Normal; Simulated limp; Simulated knee rigidity) **(I)** Self-selected **(J)** Natural clothing; Shoes condition **(K)** No load **(L)** Age; Sex; Height; Weight	**(M)** DPT; SIL; 3D-KPT (Kinect SDK) **(N)** 30 fps **(O)** DEP: 512 × 424 **(P)** Fixed single Kinect v2 (≈2.0 m height) **(Q)** Timestamped multimodal streams **(R)** Not reported
ProGait [45]	2025	4 (4 ♂)	**(A)** Indoor (clinical setting) **(B)** Dynamic background (healthcare staff present) **(C)** Planar (overground) **(D)** Controlled (mixed indoor lighting) **(E)** Multi-view (2 views: frontal; sagittal) **(F)** Straight trajectory (assisted walking) **(G)** Single-session	**(H)** Walking (Prosthetic/Pathological) **(I)** Self-selected **(J)** Natural clothing **(K)** No load **(L)** Age; Sex	**(M)** RGB; 2D-KPT (Assisted Manual Annotation); 2D-PRS **(N)** 30 fps **(O)** 1920 × 1080 **(P)** Fixed 2 cameras **(Q)** Synchronized calibrated multi-camera setup **(R)** Not reported
Scoliosis1K [20]	2024	1050 (641 ♀–409 ♂)	**(A)** Indoor (corridor) **(B)** Static background **(C)** Planar (overground) **(D)** Controlled **(E)** Single-view **(F)** Straight trajectory **(G)** Single-session	**(H)** Walking (Scoliosis screening classes: positive; neutral; negative) **(I)** Self-selected **(J)** Natural clothing **(K)** No load **(L)** Age; Sex; Height; Weight	**(M)** SIL; 2D-KPT (RTMPose) **(N)** 15 fps **(O)** 1280 × 720 **(P)** Fixed single camera (1.4–4.2 m from participants) **(Q)** Not applicable **(R)** Not reported
SPHERE Walking Dataset [16]	2014	12	**(A)** Indoor (stairs setup) **(B)** Static background **(C)** Stairs **(D)** Controlled **(E)** Single frontal view **(F)** Straight trajectory **(G)** Single-session	**(H)** Stair ascent (Normal; Left-leg lead; Right-leg lead; Freezing) **(I)** Self-selected **(J)** Natural clothing **(K)** No load **(L)** Not reported	**(M)** DPT; 3D-KPT (OpenNI) **(N)** 30 fps **(O)** 480 × 320 **(P)** Fixed single Kinect v1 **(Q)** Timestamped multimodal streams **(R)** Not reported
Walking Gait Dataset [46]	2018	9	**(A)** Indoor (laboratory setting) **(B)** Static background **(C)** Treadmill (planar) **(D)** Controlled **(E)** Single frontal view **(F)** Treadmill-constrained **(G)** Single-session	**(H)** Walking (Normal; 8 simulated asymmetric gait conditions) **(I)** Speed-controlled **(J)** Natural clothing **(K)** No load **(L)** Not reported	**(M)** SIL; 3D-KPT (Kinect SDK); 3D-PCL **(N)** 30 fps **(O)** 512 × 424 **(P)** Fixed single Kinect v2 **(Q)** Timestamped multimodal streams **(R)** Not reported

**Legend:** ♂ male; ♀ female; **(A)** Acquisition environment; **(B)** Background and scene complexity; **(C)** Walking surface; **(D)** Environmental control; **(E)** Viewpoint configuration; **(F)** Walking path or trajectory; **(G)** Temporal acquisition structure; **(H)** Activity or gait condition; **(I)** Speed or pace condition; **(J)** Clothing or appearance condition; **(K)** Carrying/load condition; **(L)** Participant descriptors; **(M)** Data modality or representation; **(N)** Frame rate or temporal sampling; **(O)** Spatial resolution; **(P)** Acquisition setup or sensor placement; **(Q)** Synchronization or alignment; **(R)** Sensor-dependent acquisition limitations. **Abbreviations:** RGB—Color Image/Video; SIL—Silhouettes; DPT—Depth Maps; 3D-PCL—3D Point Clouds; 2D-KPT—2D Keypoints; 3D-KPT—3D Keypoints; 2D-PRS—2D Human Parsing Masks; GEI—Gait Energy Image; KOA—Knee Osteoarthritis; NM—Normal; OF—Optical Flow; PD—Parkinson’s Disease; SEI—Skeleton Energy Image; AlphaPose—[47]; Kinect SDK—[48]; OpenPose—[49]; RTMPose—[50].

**Table 4:** **Biometric-oriented gait datasets** organized under the proposed covariate taxonomy. Each dataset is characterized according to scene-level ($S_{\mathrm{scene}}$), user-level ($S_{\mathrm{user}}$), and sensor-level ($S_{\mathrm{sensor}}$) factors, following the structured covariate framework (A–R) (see Section 4).

Dataset	Year	Subjects	Scene-Level	User-Level	Sensor-Level
360 Degree Gait Capture [51]	2022	65 (38 ♂–27 ♀)	**(A)** Indoor (laboratory setting); Outdoor **(B)** Static background **(C)** Planar (overground) **(D)** Uncontrolled (natural illumination; illumination changes) **(E)** Multi-view (8 views: 0°–360°; 45° increments) **(F)** Straight bidirectional walking path **(G)** Collection period reported	**(H)** Walking **(I)** Self-selected **(J)** Clothing variation **(K)** No load **(L)** Age; Sex; Height; Mass; Ethnicity	**(M)** RGB; 2D-KPT (OpenPose); GD **(N)** 30 fps **(O)** RGB: 1280 × 720 **(P)** Fixed 2 cameras (layouts varied across experiments) **(Q)** Synchronized calibrated multi-camera setup **(R)** Not reported
AVA Multi-View Dataset [52]	2013	20 (16 ♂–4 ♀)	**(A)** Indoor (studio-controlled) **(B)** Static background **(C)** Planar (overground) **(D)** Controlled **(E)** Multi-view (6 views) **(F)** Straight; Curved; Figure-eight trajectories **(G)** Single-session	**(H)** Walking **(I)** Self-selected **(J)** Natural clothing **(K)** No load **(L)** Sex	**(M)** RGB; SIL **(N)** 25 fps **(O)** 640 × 480 **(P)** Fixed 6 cameras **(Q)** Synchronized calibrated multi-camera setup **(R)** Not reported
CASIA-A [21]	2001	20	**(A)** Outdoor **(B)** Static background **(C)** Planar (overground) **(D)** Uncontrolled (natural illumination; illumination changes) **(E)** Single-view **(F)** Straight trajectories (frontal; oblique; lateral) **(G)** Single-session	**(H)** Walking **(I)** Self-selected **(J)** Natural clothing **(K)** No load **(L)** Not reported	**(M)** RGB **(N)** 25 fps **(O)** 352 × 240 **(P)** Fixed single camera **(Q)** Not applicable **(R)** Not reported
CASIA-B [25]	2005	124 (93 ♂–31 ♀)	**(A)** Indoor (laboratory setting) **(B)** Static background **(C)** Planar (overground) **(D)** Controlled **(E)** Multi-view (11 views: 0°–180°; 18° steps) **(F)** Straight trajectory **(G)** Single-session	**(H)** Walking **(I)** Self-selected **(J)** Natural clothing; Coat condition **(K)** No load; Bag **(L)** Sex; Height	**(M)** RGB; SIL **(N)** 25 fps **(O)** 320 × 240 **(P)** Fixed 11-camera arc (18° spacing) **(Q)** Not synchronized **(R)** Frame loss
CASIA-C [53]	2005	153 (130 ♂–23 ♀)	**(A)** Outdoor (night-time) **(B)** Static background **(C)** Planar (overground) **(D)** Uncontrolled (night-time thermal) **(E)** Single side view **(F)** Straight trajectory **(G)** Single-session	**(H)** Walking **(I)** Self-selected; Slow; Fast **(J)** Natural clothing **(K)** No load; Bag **(L)** Sex	**(M)** SIL; GEI; IR **(N)** 25 fps **(O)** SIL: 129 × 130; IR: 320 × 240 **(P)** Fixed single camera (thermal) **(Q)** Not applicable **(R)** Not reported
CASIA-E [26]	2022	1014 (507 ♂–507 ♀)	**(A)** Outdoor (multiple scenes) **(B)** Static background; Dynamic background **(C)** Planar (overground) **(D)** Uncontrolled (natural illumination; illumination changes) **(E)** Multi-view (26 views: 13 horizontal × 2 vertical) **(F)** Straight trajectories (multiple walking lines) **(G)** Five-month collection; seasonal variation	**(H)** Walking (stop/non-stop walking style) **(I)** Self-selected **(J)** Natural clothing; Coat condition **(K)** No load; Bag **(L)** Age; Sex; Height; Weight;Nationality	**(M)** SIL; GEI; IR **(N)** 25 fps **(O)** SIL: 1920 × 1080; IR: 640 × 480 **(P)** Fixed 8 cameras (2 heights: 1.2/3.5 m) **(Q)** Not reported **(R)** Not reported
CCGR [8]	2024	970	**(A)** Indoor (laboratory setting) **(B)** Static background **(C)** Planar; stairs; ramp; bumpy; soft; curved road **(D)** Controlled **(E)** Multi-view (33 views; 5 pitch layers) **(F)** Straight; Curved; Stairs; Ramps; Mixed task routes **(G)** 20-month collection	**(H)** Walking **(I)** Self-selected; Fast; Stationary **(J)** Natural clothing; Coat condition **(K)** No load; Book; Bag; Heavy bag; Box; Heavy box; Trolley; Umbrella **(L)** Age; Sex	**(M)** SIL; 2D-KPT (HRNet); 2D-PRS **(N)** 25 fps **(O)** 1280 × 720 **(P)** Fixed 33 cameras **(Q)** Not reported **(R)** Not reported
CCPG: Cloth-Changing benchmark for Person re-identification and Gait recognition [54]	2023	200 (122 ♂–78 ♀)	**(A)** Indoor; Outdoor **(B)** Dynamic background (static obstacles) **(C)** Planar (overground) **(D)** Uncontrolled (illumination changes) **(E)** Multi-view (10 views) **(F)** Cross-scene route; Square walking route **(G)** Single-session	**(H)** Walking **(I)** Self-selected **(J)** Clothing variation (whole-, upper-, lower-body changes) **(K)** No load; Bag **(L)** Sex	**(M)** RGB; SIL **(N)** 25 fps **(O)** RGB: 256 × 128; SIL: 128 × 88 **(P)** Fixed 10 cameras (2.7–3.0 m height) **(Q)** Not reported **(R)** Not reported
CMU Motion of Body (MoBo) [22]	2001	25 (23 ♂–2 ♀)	**(A)** Indoor (laboratory setting) **(B)** Static background **(C)** Treadmill (planar and incline) **(D)** Controlled **(E)** Multi-view (6 views) **(F)** Treadmill-constrained **(G)** Single-session	**(H)** Walking **(I)** Self-selected; Slow; Fast **(J)** Natural clothing **(K)** No load; Ball **(L)** Age; Sex; Weight	**(M)** RGB; SIL **(N)** 30 fps **(O)** 640 × 480 **(P)** Fixed 6 cameras **(Q)** Synchronized calibrated multi-camera setup **(R)** Not reported
Depth-Based Gait Dataset [55]	2015	29	**(A)** Indoor (laboratory setting) **(B)** Static background (person-induced occlusion) **(C)** Planar (overground) **(D)** Controlled **(E)** Multi-view (2 views: front, back) **(F)** Straight trajectory **(G)** Single-session	**(H)** Walking **(I)** Self-selected; Fast **(J)** Natural clothing **(K)** No load **(L)** Not reported	**(M)** SIL; DPT; 2D-KPT (Kinect SDK); 3D-KPT (Kinect SDK) **(N)** 30 fps; 15 fps **(O)** SIL: 320 × 240 **(P)** Fixed 2 Kinect v1 (2.5 m height; −27° tilt) **(Q)** Timestamped multimodal streams **(R)** Not reported
Frontal-View Gait (FVG-B) [56]	2018	226	**(A)** Outdoor **(B)** Static background (person-induced occlusion) **(C)** Planar (overground) **(D)** Uncontrolled (natural illumination) **(E)** Multi-view (3 views: −45°; 0°; +45°) **(F)** Straight trajectory **(G)** Multi-session/time gap	**(H)** Walking **(I)** Self-selected; Slow; Fast **(J)** Natural clothing; Footwear variation; Hat condition **(K)** No load; Bag **(L)** Not reported	**(M)** RGB **(N)** 15 fps **(O)** 1920 × 1080 **(P)** Fixed single camera (≈1.5 m height) **(Q)** Not applicable **(R)** Not reported
Gait3D-Parsing [30]	2023	4000	**(A)** Indoor (in-the-wild supermarket) **(B)** Dynamic background (scene-induced occlusion) **(C)** Planar (overground) **(D)** Uncontrolled (natural illumination) **(E)** Multi-view (39 views) **(F)** Unconstrained trajectories **(G)** Not reported	**(H)** Walking **(I)** Self-selected **(J)** Natural clothing **(K)** No load; Bag; Accessories **(L)** Not reported	**(M)** SIL; 2D-KPT (HRNet); 3D-KPT; 3D-MSH; 2D-PRS **(N)** 25 fps **(O)** 1920 × 1080 **(P)** Fixed 39 surveillance cameras **(Q)** Not reported **(R)** Not reported
GREW [28]	2020	26,345	**(A)** Outdoor (in-the-wild) **(B)** Dynamic background (scene-induced occlusion) **(C)** Planar (overground) **(D)** Uncontrolled (natural illumination) **(E)** Multi-view (882 views) **(F)** Unconstrained trajectories **(G)** Single-session	**(H)** Walking **(I)** Self-selected **(J)** Clothing variation **(K)** No load; Backpack; Shoulder bag; Handbag; Lift-stuff **(L)** Age; Sex	**(M)** SIL; GEI; OF; 2D-KPT (HRNet); 3D-KPT (Lifting 3D) **(N)** 25 fps **(O)** 1920 × 1080 **(P)** Fixed 882 surveillance cameras **(Q)** Not reported **(R)** Not reported
GRIDDS [13]	2018	35 (11 ♂–24 ♀)	**(A)** Indoor (laboratory setting) **(B)** Static background **(C)** Planar (overground) **(D)** Controlled **(E)** Single side view **(F)** Straight bidirectional walking path **(G)** Single-session	**(H)** Walking **(I)** Self-selected **(J)** Natural clothing **(K)** No load **(L)** Age; Sex; Height	**(M)** RGB; SIL; DPT; IR; 2D-KPT (Kinect SDK); 3D-KPT (Kinect SDK) **(N)** 30 fps **(O)** RGB: 1920 × 1080; DPT/IR: 512 × 424; SIL: 120 × 80 **(P)** Fixed single Kinect v2 (1.8 m height) **(Q)** Timestamped multimodal streams **(R)** Not reported
KY4D (A+B) [23,24]	2010–2014	42	**(A)** Indoor (laboratory setting) **(B)** Static background **(C)** Planar (overground) **(D)** Controlled **(E)** Multi-view (16 views) **(F)** Straight; Curved circular trajectories **(G)** Single-session	**(H)** Walking **(I)** Self-selected **(J)** Natural clothing **(K)** No load **(L)** Not reported	**(M)** RGB; SIL; 3D-VOL **(N)** 15 fps **(O)** 1032 × 776 **(P)** Fixed 16 circular camera setup **(Q)** Synchronized calibrated multi-camera setup **(R)** Not reported
KY IR Shadow Gait Database [57]	2014	54	**(A)** Indoor (laboratory setting) **(B)** Static background **(C)** Planar (overground) **(D)** Controlled (infrared illumination) **(E)** Multi-view (2 views: side-view; ceiling view) **(F)** Straight trajectory **(G)** Single-session	**(H)** Walking **(I)** Self-selected **(J)** Natural clothing; Down jacket; Lab coat; Coat **(K)** No load; Backpack; Hand bag; Travelling bag **(L)** Not reported	**(M)** SIL **(N)** 30 fps **(O)** 1600 × 1200 **(P)** Fixed single ceiling camera **(Q)** Not applicable **(R)** Not reported
Multi-Height Gait (MHG) [58]	2023	200	**(A)** Indoor (college gym) **(B)** Static background **(C)** Planar (overground) **(D)** Controlled **(E)** Multi-view (9 views at 45° intervals) **(F)** Straight trajectory **(G)** Single-session	**(H)** Walking **(I)** Self-selected **(J)** Natural clothing; Clothing variation **(K)** No load; Bag **(L)** Age group	**(M)** SIL **(N)** 25 fps **(O)** 128 × 88 **(P)** Fixed multi-camera setup (3 heights × 3 angles) **(Q)** Synchronized calibrated multi-camera setup **(R)** Not reported
OccGait [59]	2025	101	**(A)** Indoor (laboratory setting) **(B)** Static background (scene-induced occlusion) **(C)** Planar (overground) **(D)** Controlled **(E)** Multi-view (8 views) **(F)** Straight segments; Square walking route **(G)** Single-session	**(H)** Walking **(I)** Self-selected **(J)** Natural clothing **(K)** No load; Umbrella; Luggage **(L)** Not reported	**(M)** SIL **(N)** 25 fps **(O)** 1920 × 1080 **(P)** Fixed 3 cameras (0°; 45°; 315°) **(Q)** Synchronized calibrated multi-camera setup **(R)** Not reported
OU-ISIR Treadmill A [35]	2010	34 (26 ♂–8 ♀)	**(A)** Indoor (laboratory setting) **(B)** Static background **(C)** Treadmill (planar) **(D)** Controlled **(E)** Single side view **(F)** Treadmill-constrained **(G)** Single-session	**(H)** Walking **(I)** Speed-controlled (2–10 km/h;1 km/h steps) **(J)** Natural clothing **(K)** No load **(L)** Sex	**(M)** SIL **(N)** 60 fps **(O)** 128 × 88 **(P)** Fixed single camera **(Q)** Not applicable **(R)** Not reported
OU-ISIR Treadmill B [36]	2010	68 (31 ♂–37 ♀)	**(A)** Indoor (laboratory setting) **(B)** Static background **(C)** Treadmill (planar) **(D)** Controlled **(E)** Single side view **(F)** Treadmill-constrained **(G)** Single-session	**(H)** Walking **(I)** Not reported **(J)** Clothing variation (up to 32 clothing combinations) **(K)** No load **(L)** Age; Sex	**(M)** SIL **(N)** 60 fps **(O)** 128 × 88 **(P)** Fixed single camera **(Q)** Not applicable **(R)** Not reported
OU-ISIR Treadmill D [37]	2010	185	**(A)** Indoor (laboratory setting) **(B)** Static background **(C)** Treadmill (planar) **(D)** Controlled **(E)** Single side view **(F)** Treadmill-constrained **(G)** Single-session	**(H)** Walking **(I)** Speed-controlled **(J)** Natural clothing **(K)** No load **(L)** Not reported	**(M)** SIL **(N)** 60 fps (simulated low-FPS variants) **(O)** 128 × 88 **(P)** Fixed single camera **(Q)** Not applicable **(R)** Not reported
OU-ISIR Gait Speed Transition (GaitST) [60]	2014	179	**(A)** Indoor (laboratory setting) **(B)** Static background **(C)** Planar (overground); Treadmill (planar) **(D)** Controlled **(E)** Single side view **(F)** Treadmill-constrained **(G)** Single-session	**(H)** Walking (speed-transition) **(I)** Speed-controlled (Acceleration/De- celeration: 1–5 km/h) **(J)** Natural clothing **(K)** No load **(L)** Not reported	**(M)** SIL; GEI **(N)** 60 fps **(O)** 32 × 22 **(P)** Fixed single camera **(Q)** Not applicable **(R)** Not reported
OU-LP-Bag [29]	2018	62,528	**(A)** Indoor (museum exhibition setup) **(B)** Static background (green chroma-key) **(C)** Planar (overground) **(D)** Controlled **(E)** Single elevated side view **(F)** Straight trajectory **(G)** Long-run collection	**(H)** Walking **(I)** Self-selected **(J)** Natural clothing **(K)** No load; Bag **(L)** Age; Sex	**(M)** SIL; GEI **(N)** 25 fps **(O)** SIL: 1280 × 980; GEI: 128 × 88 **(P)** Fixed single camera (≈8 m distance; 5 m height) **(Q)** Not applicable **(R)** Not reported
OU-MVLP family [31,32,33,34]	2018–2025	10,307 (5114 ♂–5193 ♀)	**(A)** Indoor (laboratory setting) **(B)** Static background (green chroma-key) **(C)** Planar (overground) **(D)** Controlled **(E)** Multi-view (14 views: 0°–90°; 180°–270°; 15° intervals) **(F)** Straight bidirectional walking path **(G)** Long-run collection; seasonal clothing variation	**(H)** Walking **(I)** Self-selected **(J)** Natural clothing **(K)** No load **(L)** Age; Sex	**(M)** SIL; GEI; 2D-KPT (OpenPose; AlphaPose); 3D-KPT; 3D-MSH; OF **(N)** 25 fps **(O)** SIL/GEI: 128 × 88; OF: 256 × 256 **(P)** Fixed multi-camera setup (≈8 m radius; 5 m height) **(Q)** Synchronized calibrated multi-camera setup **(R)** Not reported
ReSGait [61]	2019	172 (134 ♂–38 ♀)	**(A)** Indoor (corridor) **(B)** Static background **(C)** Planar (overground) **(D)** Uncontrolled (indoor illumination) **(E)** Single-view **(F)** Straight; Curved trajectories **(G)** 15-month time span	**(H)** Walking **(I)** Self-selected **(J)** Natural clothing; Clothing variation **(K)** No load; Small item; Large item; Bag; Phone use **(L)** Sex	**(M)** SIL; 2D-KPT (OpenPose) **(N)** 20 fps **(O)** 224 × 224 **(P)** Fixed single camera (≈1.2 m height) **(Q)** Not applicable **(R)** Not reported
SAIVT-DGD [62]	2011	35	**(A)** Indoor (laboratory setting) **(B)** Static background **(C)** Planar (overground) **(D)** Controlled **(E)** Single frontal view **(F)** Straight trajectory **(G)** Single-session	**(H)** Walking **(I)** Self-selected; Fast **(J)** Natural clothing **(K)** Not reported **(L)** Not reported	**(M)** DPT; SIL; 3D-VOL **(N)** 30 fps **(O)** 640 × 480 **(P)** Fixed single Kinect v1 **(Q)** Timestamped multimodal streams **(R)** Not reported
SDUMLA-HMT [63]	2010	106 (61 ♂–45 ♀)	**(A)** Indoor (laboratory setting) **(B)** Static background **(C)** Planar (overground) **(D)** Controlled **(E)** Multi-view (6 views: 0°; 45°; 67.5°; 90°; 112.5°; 135°) **(F)** Straight trajectory **(G)** Single-session	**(H)** Walking; Running **(I)** Self-selected **(J)** Natural clothing **(K)** No load; Bag **(L)** Age; Sex	**(M)** RGB **(N)** 25 fps **(O)** 320 × 240 **(P)** Fixed 6-camera arc (radius 6 m) **(Q)** Not reported **(R)** XviD-compressed videos
SMVDU-Gait (Single/Multi) [64]	2019	20 (11 ♂–9 ♀)	**(A)** Outdoor **(B)** Static background **(C)** Planar (overground) **(D)** Uncontrolled (natural illumination) **(E)** Multi-view (3 views: lateral; oblique; frontal/back) **(F)** Straight trajectories (diagonal; front/back) **(G)** Single-session	**(H)** Walking **(I)** Self-selected **(J)** Natural clothing **(K)** No load **(L)** Age; Sex; Height; Profession	**(M)** RGB **(N)** 50 fps **(O)** 1920 × 1080 **(P)** Fixed single camera (12 m from background) **(Q)** Not applicable **(R)** Not reported
SOTON HumanID—Small DB [65]	2002	12	**(A)** Indoor (laboratory setting) **(B)** Static background **(C)** Planar (overground) **(D)** Controlled **(E)** Multi-view (4 views: side; oblique; elevated; frontal) **(F)** Straight trajectory **(G)** Single-session	**(H)** Walking **(I)** Self-selected **(J)** Clothing variation **(K)** Carrying items **(L)** Not reported	**(M)** RGB; SIL **(N)** 25 fps **(O)** 720 × 576 **(P)** Fixed multi-camera setup **(Q)** Not reported **(R)** Not reported
SOTON HumanID—Large DB [65]	2002	115 (100 ♂–15 ♀)	**(A)** Indoor; Outdoor **(B)** Static background (green chroma-key); Dynamic background **(C)** Planar (overground); Treadmill (planar) **(D)** Controlled; Uncontrolled (natural outdoor illumination) **(E)** Multi-view (6 views: fronto- parallel and oblique per scenario) **(F)** Straight bidirectional walking path; Treadmill-constrained **(G)** Single-session	**(H)** Walking **(I)** Overground: self-selected; Treadmill: constant speed **(J)** Natural clothing; Footwear **(K)** Carrying items **(L)** Sex	**(M)** RGB; SIL; OF **(N)** 25 fps **(O)** 720 × 576 **(P)** Fixed multi-camera setup **(Q)** Camera sync information available **(R)** Not reported
SUSTech1K [27]	2023	1050	**(A)** Outdoor (multiple scenes) **(B)** Dynamic background (scene-induced occlusion) **(C)** Planar (overground) **(D)** Uncontrolled (natural illumination;day/night variation) **(E)** Multi-view (12 views) **(F)** Straight; Round-trip with turns **(G)** Multi-session (day/night variation)	**(H)** Walking **(I)** Self-selected **(J)** Natural clothing; Clothing variation **(K)** Bag; Object carrying; Umbrella **(L)** Not reported	**(M)** RGB; SIL; 3D-PCL **(N)** RGB: 30 fps; LiDAR: 10 fps **(O)** RGB: 1280 × 980; SIL: 64 × 64 **(P)** Mobile single robot-mounted RGB–LiDAR system **(Q)** Timestamped multimodal frames; GPS-clock synchronized **(R)** LiDAR noise
SZU RGB-D Gait Dataset [66]	2013	99	**(A)** Indoor **(B)** Static background **(C)** Planar (overground) **(D)** Controlled **(E)** Multi-view (2 views: 90° side; ≈60° oblique) **(F)** Straight trajectories (side; diagonal) **(G)** Single-session	**(H)** Walking **(I)** Self-selected **(J)** Natural clothing **(K)** No load **(L)** Not reported	**(M)** RGB; DPT; 3D-PCL; SIL; GEI **(N)** 30 fps **(O)** 640 × 480 **(P)** Fixed single ASUS Xtion PRO LIVE (≈80 cm height) **(Q)** RGB–depth calibrated/aligned **(R)** Not reported
TUM-IITKGP [67]	2010	35	**(A)** Indoor (corridor) **(B)** Static background (static and dynamic inter-object occlusion) **(C)** Planar (overground) **(D)** Controlled (indoor illumination) **(E)** Single side view **(F)** Straight bidirectional walking path **(G)** Single-session	**(H)** Walking **(I)** Self-selected **(J)** Natural clothing; Clothing variation **(K)** No load; Backpack **(L)** Not reported	**(M)** SIL **(N)** 30 fps **(O)** 640 × 480 **(P)** Fixed single camera (≈1.85 m height) **(Q)** Not applicable **(R)** Not reported

**Legend:** ♂ male; ♀ female; **(A)** Acquisition environment; **(B)** Background and scene complexity; **(C)** Walking surface; **(D)** Environmental control; **(E)** Viewpoint configuration; **(F)** Walking path or trajectory; **(G)** Temporal acquisition structure; **(H)** Activity or gait condition; **(I)** Speed or pace condition; **(J)** Clothing or appearance condition; **(K)** Carrying/load condition; **(L)** Participant descriptors; **(M)** Data modality or representation; **(N)** Frame rate or temporal sampling; **(O)** Spatial resolution; **(P)** Acquisition setup or sensor placement; **(Q)** Synchronization or alignment; **(R)** Sensor-dependent acquisition limitations. **Abbreviations:** RGB—Color Image/Video; SIL—Silhouettes; DPT—Depth Maps; 3D-PCL—3D Point Clouds; 2D-KPT—2D Keypoints; 3D-KPT—3D Keypoints; 2D-PRS—2D Human Parsing Masks; GD—Goniometer Data; GEI—Gait Energy Image; KOA—Knee Osteoarthritis; NM—Normal; OF—Optical Flow; PD—Parkinson’s Disease; SEI—Skeleton Energy Image; AlphaPose—[47]; HRNet—[68]; Lifting 3D—[69]; Kinect SDK—[48]; MotionBERT—[70]; OpenPose—[49]; RTMPose—[50].

**Table 5:** **Attribute-recognition-oriented gait datasets** organized under the proposed covariate taxonomy. Each dataset is characterized according to scene-level ($S_{\mathrm{scene}}$), user-level ($S_{\mathrm{user}}$), and sensor-level ($S_{\mathrm{sensor}}$) factors, following the structured covariate framework (A–R) (see Section 4).

Dataset	Year	Subjects	Scene-Level	User-Level	Sensor-Level
MA-Gait [38]	2022	95 (65 ♂–30 ♀)	**(A)** Indoor **(B)** Static background **(C)** Planar (overground) **(D)** Controlled **(E)** Multi-view (12 views: 0°–360°; 30° intervals) **(F)** Straight bidirectional walking path **(G)** Single-session	**(H)** Walking (12 patterns; 16 attribute labels) **(I)** Self-selected **(J)** Natural clothing **(K)** No load **(L)** Age; Sex	**(M)** SIL; 2D-KPT (HRNet) **(N)** 25 fps **(O)** RGB: 1920 × 1080; SIL: 64 × 44 **(P)** Fixed 6 cameras (2.2 m height) **(Q)** Not reported **(R)** Not reported
OU-LP-Age [40]	2017	63,846 (31,093 ♂–32,753 ♀)	**(A)** Indoor **(B)** Static background (green chroma-key) **(C)** Planar (overground) **(D)** Controlled **(E)** Single side view **(F)** Straight trajectory **(G)** Long-run exhibition collection	**(H)** Walking **(I)** Self-selected **(J)** Natural clothing **(K)** No load **(L)** Age; Sex	**(M)** GEI **(N)** 30 fps **(O)** 128 × 88 **(P)** Fixed single camera (≈4 m from walking course) **(Q)** Not applicable **(R)** Not reported
RA-GAR [39]	2025	533	**(A)** Outdoor **(B)** Dynamic background **(C)** Planar (overground) **(D)** Uncontrolled (natural illumination; illumination changes) **(E)** Multi-view (10 views: 0°–360°; 36° intervals) **(F)** Straight trajectory **(G)** Single-session	**(H)** Walking (15 gait attributes) **(I)** Self-selected **(J)** Clothing variation **(K)** No load; Backpack **(L)** Age; Sex; Height; Body mass	**(M)** SIL; 2D-KPT (RTMPose); 3D-KPT (MotionBERT) **(N)** 30 fps **(O)** RGB: 1920 × 1080; SIL: 64 × 44 **(P)** Fixed 5 cameras (1.3 m height) **(Q)** Not reported **(R)** Not reported

**Legend:** ♂ male; ♀ female; **(A)** Acquisition environment; **(B)** Background and scene complexity; **(C)** Walking surface; **(D)** Environmental control; **(E)** Viewpoint configuration; **(F)** Walking path or trajectory; **(G)** Temporal acquisition structure; **(H)** Activity or gait condition; **(I)** Speed or pace condition; **(J)** Clothing or appearance condition; **(K)** Carrying/load condition; **(L)** Participant descriptors; **(M)** Data modality or representation; **(N)** Frame rate or temporal sampling; **(O)** Spatial resolution; **(P)** Acquisition setup or sensor placement; **(Q)** Synchronization or alignment; **(R)** Sensor-dependent acquisition limitations. **Abbreviations:** RGB—Color Image/Video; SIL—Silhouettes; 2D-KPT—2D Keypoints; 3D-KPT—3D Keypoints; GEI—Gait Energy Image; HRNet—[68]; MotionBERT—[70]; RTMPose—[50].

## Data Availability

The original contributions presented in this study are included in the article. Further inquiries can be directed to the corresponding author.

## Supplementary Materials

The following supporting information can be downloaded at: https://www.mdpi.com/article/10.3390/jimaging12070334/s1, PRISMA 2020 Checklist.

## Appendix A. List of Included Datasets

Appendix A provides the complete list of datasets included in this review, grouped by main area of use and accompanied by access information. Table A1 presents healthcare-oriented datasets, Table A2 presents biometric-oriented datasets, and Table A3 presents attribute-recognition datasets. The repository links and access routes are provided to support reproducibility in the digital version of the article and were verified during manuscript preparation. Because dataset repositories, institutional webpages, and access procedures may change over time, these links should be interpreted as access information valid at the time of writing.

**Table A1:** Healthcare-oriented gait datasets included in this review, with official access information and access dates.

Dataset	Year	URL	Access Date
Gait-A Database [43]	2016	http://hdl.handle.net/10045/70567	1 June 2026
GAIT-IST [14]	2020	http://www.img.lx.it.pt/GAIT-IST/	1 June 2026
GAIT-IT [17]	2021	http://www.img.lx.it.pt/GAIT-IT/	1 June 2026
Health&Gait [19]	2025	https://zenodo.org/records/14039922	1 June 2026
INIT Gait Database [15]	2016	https://www.vision.uji.es/gaitDB/	1 June 2026
KOA-PD-NM [18]	2020	https://data.mendeley.com/datasets/44pfnysy89/1	1 June 2026
MMGS [44]	2019	https://github.com/margokhokhlova/LSTM_gait_model	1 June 2026
ProGait [45]	2025	https://huggingface.co/datasets/ericyxy98/ProGait	1 June 2026
Scoliosis1K Dataset [20]	2024	https://zhouzi180.github.io/Scoliosis1K/	1 June 2026
SPHERE Walking Dataset [16]	2014	https://doi.org/10.5523/bris.bgresiy3olk41nilo7k6xpkqf	1 June 2026
Walking Gait Dataset [46]	2018	https://www-labs.iro.umontreal.ca/~labimage/GaitDataset/	1 June 2026

**Table A2:** Biometric-oriented gait datasets included in this review, with official access information and access dates.

Dataset	Year	URL	Access Date
360 Degree Gait Capture [51]	2022	https://doi.org/10.24377/LJMU.d.00000133	1 June 2026
AVA Multi-View Dataset [52]	2013	https://www.uco.es/investiga/grupos/ava/portfolio/avamvg/	1 June 2026
CASIA-A [21]	2001	http://www.cbsr.ia.ac.cn/english/Gait%20Databases.asp	1 June 2026
CASIA-B [25]	2005	http://www.cbsr.ia.ac.cn/english/Gait%20Databases.asp	1 June 2026
CASIA-C [53]	2005	http://www.cbsr.ia.ac.cn/english/Gait%20Databases.asp	1 June 2026
Multi-Height Gait [58]	2023	https://housaihui.cn/TBIOM-Metric-Gait/	1 June 2026
OccGait [59]	2025	https://github.com/BNU-IVC/OccGait	1 June 2026
OU-ISIR Treadmill A [35]	2010	http://www.am.sanken.osaka-u.ac.jp/BiometricDB/GaitTM.html	1 June 2026
OU-ISIR Treadmill B [36]	2010	http://www.am.sanken.osaka-u.ac.jp/BiometricDB/GaitTM.html	1 June 2026
OU-ISIR Treadmill D [37]	2010	http://www.am.sanken.osaka-u.ac.jp/BiometricDB/GaitTM.html	1 June 2026
OU-ISIR GST [60]	2014	http://www.am.sanken.osaka-u.ac.jp/BiometricDB/GaitST.html	1 June 2026
OU-ISIR LP Bag [29]	2018	http://www.am.sanken.osaka-u.ac.jp/BiometricDB/GaitLPBag.html	1 June 2026
CASIA-E [26]	2022	https://doi.org/10.57760/sciencedb.07226	1 June 2026
CCPG [54]	2023	https://github.com/BNU-IVC/CCPG	1 June 2026
CMU Motion of Body [22]	2001	https://www.ri.cmu.edu/publications/the-cmu-motion-of-body-mobo-database/	1 June 2026
CCGR [8]	2024	https://github.com/ShinanZou/CCGR	1 June 2026
Depth-Based Gait Dataset [55]	2015	https://facweb.iitkgp.ac.in/~shamik/Gait/Dataset1.html	1 June 2026
Frontal-View Gait [56]	2018	https://cvlab.cse.msu.edu/frontal-view-gaitfvg-database.html	1 June 2026
Gait3D-Parsing [30]	2023	https://gait3d.github.io/gait3d-parsing-hp/	1 June 2026
GREW [28]	2020	http://www.grew-benchmark.org/index.html	1 June 2026
GRIDDS [13]	2018	http://gridds.ipvc.pt/	1 June 2026
KY4D (A + B) [23,24]	2010	https://robotics.ait.kyushu-u.ac.jp/yumi/db.html	1 June 2026
KY IR [57]	2014	https://robotics.ait.kyushu-u.ac.jp/yumi/db/ir_shadow.html	1 June 2026
OU-ISIR LP MV [31,32,33,34]	2018–2025	http://www.am.sanken.osaka-u.ac.jp/BiometricDB/GaitMVLP.html	1 June 2026
ReSGait [61]	2019	https://github.com/zihaomu/resgait	1 June 2026
SAIVT-Depth Gait Database [62]	2011	https://research.qut.edu.au/saivt/databases/saivt-dgd-database/	1 June 2026
SDUMLA-HMT [63]	2010	http://www.sai.sdu.edu.cn/info/1075/1115.htm	1 June 2026
SMVDU-Gait [64]	2019	https://data.mendeley.com/datasets/py4zw6g7xc/3	1 June 2026
SOTON HumanID—Small/Large [65]	2002	https://web-archive.southampton.ac.uk/www.gait.ecs.soton.ac.uk/database/large_db.php3.html	1 June 2026
SUSTech1K [27]	2023	https://lidargait.github.io/	1 June 2026
SZU RGB-D Gait [66]	2013	http://pan.baidu.com/s/1gdGQ3EN	1 June 2026
TUM-IITKGP [67]	2010	https://www.ce.cit.tum.de/mmk/verschiedenes/tum-iitkgp-gait-database/	1 June 2026

**Table A3:** Attribute-recognition gait datasets included in this review, with official access information and access dates.

Dataset	Year	URL	Access Date
Multi-Attribute Gait [38]	2022	https://doi.org/10.1109/TIFS.2023.3318934	1 June 2026
OU-ISIR LP Age [40]	2017	http://www.am.sanken.osaka-u.ac.jp/BiometricDB/GaitLPAge.html	1 June 2026
RA-GAR [39]	2025	https://github.com/BNU-IVC/RA-GAR	1 June 2026
