# Supplementary material for: Structuring Variability in Human Gait Datasets: A Covariate-Centered Taxonomy and Systematic Review of Image- and Depth-Based Collections
Source: J Imaging. 2026 Jul 22;12(7):334. doi: 10.3390/jimaging12070334 (PMC13412367; doi:10.3390/jimaging12070334)
Supplement: Supplementary file 1 [file jimaging-12-00334-s001.zip › jimaging-4389877-supplementary.pdf]

PRISMA 2020 Checklist

Manuscript: *Structuring Variability in Human Gait Datasets: A Covariate-Centered Taxonomy and Systematic Review of Image- and Depth-Based Collections*

Journal of Imaging manuscript ID: jimaging-4389877

Authors: João Ferreira Nunes, Pedro Miguel Moreira, and João Manuel R. S. Tavares

This completed checklist maps each PRISMA 2020 item to the location where it is addressed in the current manuscript version. Items marked as *Not applicable.* reflect the dataset-oriented and descriptive nature of the review, for which no intervention effects, meta-analysis, effect estimates, or certainty-of-evidence grading were performed. Items marked as *Partially reported.* indicate that the manuscript contains relevant information but does not report every detail requested by the PRISMA item.

Table S1: Completed PRISMA 2020 checklist for the submitted systematic review.

Section and Topic	Item #	Checklist item	Location where item is reported
**TITLE**			
Title	1	Identify the report as a systematic review.	Title page. The title includes "Systematic Review"; the manuscript type is also set as a systematic review.
**ABSTRACT**			
Abstract	2	See the PRISMA 2020 for Abstracts checklist.	Abstract. The abstract reports the review context, taxonomy objective, PRISMA-aligned protocol, number of included datasets, application domains, and main descriptive findings. A separate PRISMA-for-Abstracts checklist is not included in this document.
**INTRODUCTION**			
Rationale	3	Describe the rationale for the review in the context of existing knowledge.	Section 1, *Introduction*; Table 1, comparison with prior surveys and taxonomies.
Objectives	4	Provide an explicit statement of the objective(s) or question(s) the review addresses.	Section 1, *Introduction*; Section 1.1, *Contributions*; Section 2.1, *Research Questions*.
**METHODS**			
Eligibility criteria	5	Specify the inclusion and exclusion criteria for the review and how studies were grouped for the syntheses.	Section 2.3, *Eligibility Criteria*; Section 3, *Systematic Review by Main Area of Use*, which defines healthcare-oriented, biometric-oriented, and attribute-recognition groupings; Section 5, taxonomy-aligned tables by domain.
Information sources	6	Specify all databases, registers, websites, organisations, reference lists and other sources searched or consulted to identify studies. Specify the date when each source was last searched or consulted.	Section 2.2, *Search Strategy*, reports IEEE Xplore, Scopus, Web of Science, Google Scholar, backward/forward snowballing, official dataset webpages, institutional repositories, GitHub repositories, and dataset hosting platforms. Appendix A, Tables A1–A3, reports dataset access URLs and access dates. Exact last-search dates for each bibliographic database are not separately reported.
Search strategy	7	Present the full search strategies for all databases, registers and websites, including any filters and limits used.	Section 2.2, *Search Strategy*, reports the general Boolean search structures, publication window (2000–2025), and complementary query variants.
Selection process	8	Specify the methods used to decide whether a study met the inclusion criteria of the review, including how many reviewers screened each record and each report retrieved, whether they worked independently, and if applicable, details of automation tools used in the process.	Section 2.4, *Screening and Selection Process*; Figure 1, adapted PRISMA 2020-style flow diagram. The manuscript reports multi-stage screening, full-text eligibility assessment, consolidation of duplicate dataset reports, and triangulation of dataset paper/documentation/access page. Two independent screeners were involved and no automation-tool was used.

Continued on next page

Section and Topic	Item #	Checklist item	Location where item is reported
Data collection process	9	Specify the methods used to collect data from reports, including how many reviewers collected data from each report, whether they worked independently, any processes for obtaining or confirming data from study investigators, and if applicable, details of automation tools used in the process.	Section 2.5, *Data Extraction and Taxonomy Coding*; Author Contributions, where investigation, data curation, and formal analysis are attributed to J.F.N. The manuscript reports structured extraction and triangulation across dataset papers, official documentation, repositories, and access pages.
Data items	10a	List and define all outcomes for which data were sought. Specify whether all results that were compatible with each outcome domain in each study were sought and, if not, the methods used to decide which results to collect.	*Not applicable.* This is a dataset-oriented review rather than an intervention/effect review. No clinical or experimental outcome effects were sought. Review questions and dataset-level analytical objectives are reported in Section 2.1, *Research Questions*.
Data items	10b	List and define all other variables for which data were sought. Describe any assumptions made about any missing or unclear information.	Section 2.5, *Data Extraction and Taxonomy Coding*; Section 4, *Covariate Taxonomy Framework*, defining covariates A–R; Section 5, taxonomy-aligned tables. Missing or unclear variables were coded conservatively as not reported.
Study risk of bias assessment	11	Specify the methods used to assess risk of bias in the included studies, including details of the tool(s) used, how many reviewers assessed each study and whether they worked independently, and if applicable, details of automation tools used in the process.	*Not applicable.* No formal study risk-of-bias tool was applied because the unit of analysis is the dataset, not an intervention or diagnostic-effect study. Section 2.6, *Dataset Quality, Access, and Documentation Assessment*, reports a qualitative, descriptive assessment of reusability, documentation, access, annotation traceability, and protocol reporting.
Effect measures	12	Specify for each outcome the effect measure(s) used in the synthesis or presentation of results.	*Not applicable.* No effect measures such as risk ratios, mean differences, or confidence intervals were used. The manuscript reports descriptive dataset counts, percentages, and covariate coverage summaries in Section 6 and Figures 3–5.
Synthesis methods	13a	Describe the processes used to decide which studies were eligible for each synthesis.	Section 2.3, *Eligibility Criteria*; Section 2.4, *Screening and Selection Process*; Section 3, domain grouping logic; Section 5, taxonomy-aligned tables grouped by main area of use.
Synthesis methods	13b	Describe any methods required to prepare the data for presentation or synthesis, such as handling of missing summary statistics, or data conversions.	Section 2.5, *Data Extraction and Taxonomy Coding*; Section 4, *Covariate Taxonomy Framework*; Section 4.6, *Boundary Cases and Coding Rules*; Section 5, A–R coding tables. Missing or undocumented covariates were coded as not reported rather than inferred.
Synthesis methods	13c	Describe any methods used to tabulate or visually display results of individual studies and syntheses.	Section 5, taxonomy-aligned dataset tables; Section 6, analytical insights; Figures 3–5, covariate value distributions; Appendix A, Tables A1–A3, included datasets with access information.
Synthesis methods	13d	Describe any methods used to synthesize results and provide a rationale for the choice(s). If meta-analysis was performed, describe the model(s), method(s) to identify statistical heterogeneity, and software package(s) used.	Section 6, *Analytical Insights Enabled by the Taxonomy*; Section 7, *Structural Gaps and Research Implications.* The synthesis is descriptive and taxonomy-based; no meta-analysis was performed.

Continued on next page

Section and Topic	Item #	Checklist item	Location where item is reported
Synthesis methods	13e	Describe any methods used to explore possible causes of heterogeneity among study results.	Section 6, domain-stratified covariate patterns and covariate value distributions; Section 7, structural gap analysis. Heterogeneity is explored descriptively across application domains, acquisition settings, user-level covariates, and sensor-level reporting. No subgroup meta-analysis or meta-regression was conducted.
Synthesis methods	13f	Describe any sensitivity analyses conducted to assess robustness of the synthesized results.	*Not applicable.* No sensitivity analyses were conducted or reported.
Reporting bias assessment	14	Describe any methods used to assess risk of bias due to missing results in a synthesis.	*Not applicable.* No formal reporting-bias assessment was conducted. Relevant reporting and availability limitations are addressed descriptively in Section 2.6 and Section 7.9–7.10, focusing on documentation quality, access stability, annotation traceability, and standardized reporting.
Certainty assessment	15	Describe any methods used to assess certainty or confidence in the body of evidence for an outcome.	*Not applicable.* No certainty-of-evidence assessment was conducted because the review does not synthesize effect estimates or outcome-level intervention evidence.
**RESULTS**			
Study selection	16a	Describe the results of the search and selection process, from the number of records identified in the search to the number of studies included in the review, ideally using a flow diagram.	Section 2.4, *Screening and Selection Process*; Figure 1, PRISMA-style flow diagram. Reported counts: 986 records identified, 244 duplicates removed, 742 records screened, 624 excluded after title/abstract screening, 118 full-text sources assessed, 71 excluded or consolidated, 47 datasets included.
Study selection	16b	Cite studies that might appear to meet the inclusion criteria, but which were excluded, and explain why they were excluded.	*Partially reported.* Figure 1 reports main exclusion/consolidation reasons. Section 2.4 explains exclusion logic for non-eligible modality/task scope, insufficient documentation, non-reproducible access, and derivative/duplicate dataset reports. Individual excluded studies that might appear eligible are not cited in a separate exclusion list.
Study characteristics	17	Cite each included study and present its characteristics.	Section 3, *Systematic Review by Main Area of Use*; Section 5, taxonomy-aligned Tables 3–5; Appendix A, Tables A1–A3, complete list of included datasets with access information.
Risk of bias in studies	18	Present assessments of risk of bias for each included study.	*Not applicable.* Formal risk-of-bias assessments were not performed for individual datasets. Dataset quality, access, annotation traceability, documentation quality, and reporting limitations are discussed descriptively in Section 2.6 and Section 7.9–7.10.
Results of individual studies	19	For all outcomes, present, for each study: summary statistics for each group and effect estimate and its precision, ideally using structured tables or plots.	*Not applicable.* No outcome effects or precision estimates were synthesized. Dataset-level characteristics are presented in structured tables: Section 5, Tables 3–5; Appendix A, Tables A1–A3.

Continued on next page

Section and Topic	Item #	Checklist item	Location where item is reported
Results of syntheses	20a	For each synthesis, briefly summarise the characteristics and risk of bias among contributing studies.	*Partially reported.* Section 6 summarizes characteristics and covariate patterns across included datasets and application domains. Risk of bias is not formally assessed; documentation and reusability limitations are discussed descriptively in Section 7.
Results of syntheses	20b	Present results of all statistical syntheses conducted. If meta-analysis was done, present the summary estimate and its precision and measures of statistical heterogeneity. If comparing groups, describe the direction of the effect.	Section 6, descriptive quantitative synthesis using counts and percentages; Figures 3–5, covariate value distributions. No meta-analysis or inferential statistical synthesis was conducted.
Results of syntheses	20c	Present results of all investigations of possible causes of heterogeneity among study results.	Section 6, scene-level, user-level, and sensor-level patterns; Section 7, structural gaps and research implications by covariate category.
Results of syntheses	20d	Present results of all sensitivity analyses conducted to assess the robustness of the synthesized results.	*Not applicable.* No sensitivity analyses were conducted or reported.
Reporting biases	21	Present assessments of risk of bias due to missing results arising from reporting biases for each synthesis assessed.	*Not applicable.* No formal reporting-bias assessment was conducted. Missing or incomplete reporting is addressed descriptively in Section 2.5, Section 2.6, and Section 7.9–7.10.
Certainty of evidence	22	Present assessments of certainty or confidence in the body of evidence for each outcome assessed.	*Not applicable.* No certainty assessment was conducted because no outcome-level body of evidence or effect synthesis was assessed.
**DISCUSSION**			
Discussion	23a	Provide a general interpretation of the results in the context of other evidence.	Section 6, *Analytical Insights Enabled by the Taxonomy*; Section 7, *Structural Gaps and Research Implications*; Section 8, *Conclusions*.
Discussion	23b	Discuss any limitations of the evidence included in the review.	Section 7, especially Sections 7.1–7.10, discussing ecological diversity, viewpoint coverage, temporal protocols, demographics, modalities, synchronization/alignment, sensor limitations, access, annotation traceability, and reporting standardization.
Discussion	23c	Discuss any limitations of the review processes used.	Section 2.5 reports conservative coding of unclear or unreported covariates; Appendix A notes that repository links and access procedures may change over time.
Discussion	23d	Discuss implications of the results for practice, policy, and future research.	Section 7, *Structural Gaps and Research Implications*; Section 8, *Conclusions*.
**OTHER INFORMATION**			
Registration and protocol	24a	Provide registration information for the review, including register name and registration number, or state that the review was not registered.	Section 2, *Review Methodology*. The review protocol is reported as registered in the Open Science Framework (OSF) with DOI https://doi.org/10.17605/OSF.IO/MSNZY.

Continued on next page

Section and Topic	Item #	Checklist item	Location where item is reported
Registration and protocol	24b	Indicate where the review protocol can be accessed, or state that a protocol was not prepared.	Section 2, *Review Methodology*; OSF DOI https://doi.org/10.17605/OSF.IO/MSNZY; reference to Nunes et al. OSF registration.
Registration and protocol	24c	Describe and explain any amendments to information provided at registration or in the protocol.	*Not reported.* No amendments to the OSF registration/protocol are described in the manuscript.
Support	25	Describe sources of financial or non-financial support for the review, and the role of the funders or sponsors in the review.	Funding statement: "This research received no external funding." No funder or sponsor role is reported.
Competing interests	26	Declare any competing interests of review authors.	Conflicts of Interest statement: "The authors declare no conflicts of interest."
Availability of data, code and other materials	27	Report which review materials are publicly available and where they can be found: template data collection forms, extracted data, data used for analyses, analytic code, and other materials.	*Partially reported.* Data Availability Statement: original contributions are included in the article and inquiries can be directed to the corresponding author. Section 5 and Appendix A provide extracted dataset characteristics, taxonomy coding, access URLs, and access dates. Template data collection forms, analytic code, and a separately deposited extracted-data file are not reported as publicly available.

Checklist source. Adapted from Page MJ, McKenzie JE, Bossuyt PM, Boutron I, Hoffmann TC, Mulrow CD, et al. *The PRISMA 2020 statement: an updated guideline for reporting systematic reviews.* BMJ 2021;372:n71. doi: 10.1136/bmj.n71. The PRISMA 2020 checklist is licensed under CC BY 4.0.
